# Experimental evolution reveals bifunctional genetic solutions to loss of *trpF* in *Salmonella enterica*

**DOI:** 10.1093/g3journal/jkag167

**Published:** 2026-07-06

**Authors:** Joakim Näsvall, Hind Abdalaal

**Affiliations:** Department of Medical Biochemistry and Microbiology, Uppsala University, Uppsala 751 23, Sweden; Department of Medical Biochemistry and Microbiology, Uppsala University, Uppsala 751 23, Sweden

**Keywords:** intermittent selection, adaptive conflict, evolutionary rescue, bifunctional genes, noncanonical translation, adaptive mutations

## Abstract

How new gene functions arise while maintaining ancestral biological roles remains a central question in evolutionary genetics. To investigate genetic solutions to the disruption of a biosynthetic pathway without prior genetic bias, we used experimental evolution to study restoration of tryptophan biosynthesis in *Salmonella enterica* strains lacking the *trpF* gene. Populations were founded from bacteria carrying wild-type alleles of all relevant genes in their native genomic and regulatory contexts and evolved under conditions selecting for growth without exogenous tryptophan. Across independent populations, mutations in either *hisA* or *trpA* enabled growth of Δ*trpF* strains in the absence of added tryptophan while retaining sufficient ancestral function to support growth under the same conditions. Whole-genome sequencing and genetic reconstruction showed that these mutant alleles were sufficient to confer the growth-rescue phenotype. Duplication of the target gene was detected in only a single population and showed no evidence of functional divergence. Mutational paths differed between genes: *hisA*-based solutions arose primarily in mutator backgrounds and were associated with stronger trade-offs with ancestral function, whereas *trpA*-based solutions were more frequent and often retained native function. Together, these results demonstrate that bifunctional genetic solutions can arise through point mutations in multiple genes during experimental adaptation, illustrating how physiologically relevant multifunctionality can evolve at the gene level without gene duplication.

## Graphical Abstract

**Graphical Abstract:**
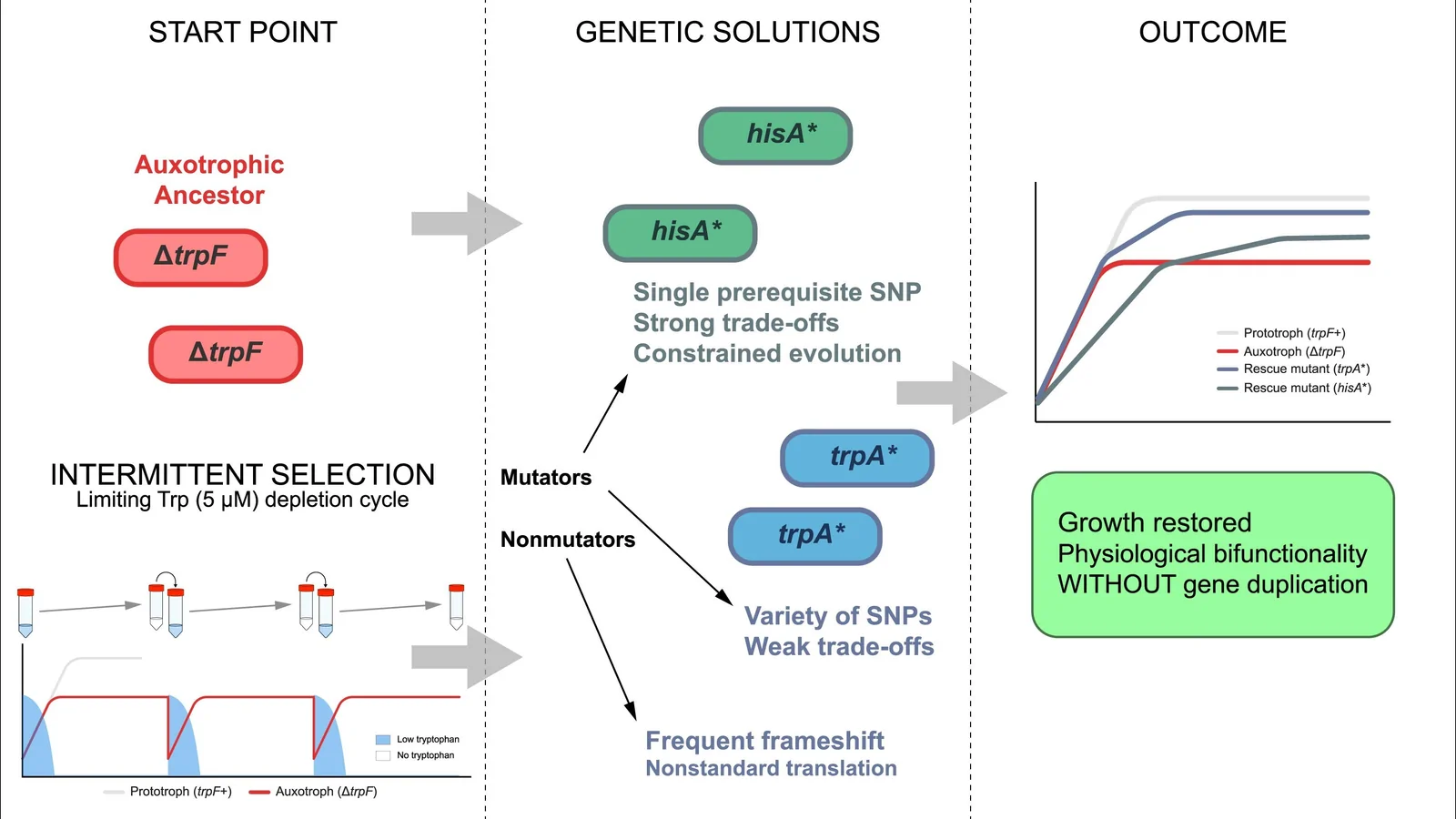

## Introduction

The evolution of new gene functions often involves mutations that alter existing biological roles, creating trade-offs between ancestral and newly acquired activities (Soskine and Tawfik 2010). Such conflicts can be resolved through gene duplication followed by divergence (Ohno 1970; Bergthorsson et al. 2007; Deng et al. 2010; Näsvall et al. 2012). However, although relatively rare in the literature, some genes in nature perform more than one physiologically relevant function, indicating that multifunctionality can also arise and persist without duplication (Mueller et al. 1998; Barona-Gómez and Hodgson 2003). Understanding how genes acquire additional functions while retaining ancestral roles is therefore central to evolutionary genetics, but direct experimental evidence linking specific genetic changes to such outcomes remains limited (Näsvall et al. 2012; Lundin et al. 2020).

One experimental system that has proven particularly informative for studying the evolution of new gene functions involves the histidine and tryptophan biosynthetic pathways in bacteria. In many bacteria, the enzymes HisA and TrpF catalyze chemically related reactions on structurally similar substrates (Supplementary Fig. 1), and both belong to the same family of (αβ)_8_-barrel enzymes, reflecting a shared evolutionary origin (Copley and Bork 2000). Consistent with this relationship, previous work has shown that HisA can acquire TrpF activity through mutation, often accompanied by trade-offs with its native function (Jürgens et al. 2000; Näsvall et al. 2012; Newton et al. 2017; Lundin et al. 2020). In addition, some actinobacterial species lack a dedicated *trpF* gene and instead rely on a single HisA ortholog, PriA, that supports both histidine and tryptophan biosynthesis, demonstrating that physiologically relevant bifunctionality can be maintained in nature (Barona-Gómez and Hodgson 2003; Due et al. 2011; Plach et al. 2016). Additionally, TrpA, an enzyme that acts later in the tryptophan biosynthetic pathway (Supplementary Fig. 1) and is less closely related to TrpF, has also been shown to acquire TrpF activity through directed evolution and rational mutagenesis, indicating that multiple genes can, in principle, provide genetic solutions to loss of *trpF* (Evran et al. 2012).

To reduce experimental biases that can influence which genetic solutions are observed during adaptation, we designed an experimental evolution framework starting from minimally modified genetic backgrounds. We used *Salmonella enterica* strains lacking *trpF* but otherwise carrying wild-type alleles of *hisA*, *trpA*, and all other relevant genes in their native genomic locations and under their native regulation. Because deletion of *trpF* prevents growth in the absence of tryptophan, populations were propagated in medium containing a limiting amount of tryptophan, allowing initial growth of the ancestral genotype while selecting for restoration of tryptophan biosynthesis as the nutrient became depleted (Fig. 1). This fluctuating, intermittent selection regime could serve as a functional analog to models of microbial range expansion or niche invasion, where populations at an expanding pioneering front can experience a transition from locally abundant resources to nutrient depletion (Bosshard et al. 2019). This approach differs from previous studies that relied on engineered starting alleles, altered gene expression, genomic contexts prone to amplification, or targeted mutagenesis, all of which can bias evolutionary trajectories toward particular genes or mechanisms (Jürgens et al. 2000; Evran et al. 2012; Näsvall et al. 2012; Lundin et al. 2020).

Using this framework, we followed the early stages of adaptation across replicate populations and identified the genetic changes that enabled growth of Δ*trpF* strains without added tryptophan. Whole-genome sequencing and reconstruction of evolved alleles were then used to determine which mutations were sufficient to confer the growth-rescue phenotype, allowing us to characterize the genetic basis and functional consequences of the adaptations that emerged.

## Materials and methods

### Bacterial strains and growth conditions

All bacterial strains used in this study (listed in Supplementary Table 1) are derivatives of *Salmonella enterica* subsp. *enterica* serovar Typhimurium strain LT2, except for 3 *Escherichia coli* strains used as PCR templates for amplification of selection cassettes employed in λ Red recombineering. Unless otherwise stated, generalized transduction was performed using phage P22 *HT105*/*1 int*-*201* (Schmieger 1972) or the defective P22-derived artificial gene transfer agent GTA22 (described below).

Lysogeny broth (LB; 10 g/L tryptone, 5 g/L yeast extract, 10 g/L NaCl) was used as a rich medium, with 15 g/L Bacto agar added for solid medium (LA). Salt-free LB (LB without NaCl) was used for preparing electrocompetent cells. SOC medium (Hanahan 1983) was used for recovery following electroporation. As a minimal medium, M9 medium (Miller 1992) was prepared at double strength with respect to M9 salts, glucose, CaCl_2_, and MgCl_2_ (hereafter referred to as 2× M9) to allow growth to higher cell densities. Where indicated, minimal medium was supplemented with tryptophan (0.1 mM or 5 μM), histidine (0.1 mM), and/or guanosine (0.3 mM; See Supplementary Results and Discussion). Solid M9 plates contained 15 g/L Bacto agar. All cultures were grown at 37 °C except during λ Red recombineering.

Antibiotics were used only for the selection of recombinants following genetic manipulations and included trimethoprim (10 mg/L), tetracycline (7.5 mg/L), and chloramphenicol (12.5 mg/L).

### Genetic manipulations and strain construction

Genetic markers, deletions, and allelic replacements were constructed using λ Red recombineering as described previously (Yu et al. 2000; Näsvall 2017; Näsvall et al. 2017; Näsvall 2022). Strains carrying the λ Red helper plasmid pSIM5-Tet (Koskiniemi et al. 2011) were grown overnight at 30 °C in salt-free LB supplemented with 0.2% glucose, diluted 1:100, and induced for recombination by incubation at 42 °C for 15 min. Cells were rendered electrocompetent by washing in ice-cold water or 10% glycerol and electroporated with purified PCR products.

Mutations in *hisA* and *trpA* were reconstructed by PCR amplification of the mutated alleles from evolved populations or clones, followed by replacement of a selectable and counter-selectable cassette (*Atox1*; GenBank MN207489) inserted at the target locus. Selection for loss of the cassette was performed on M9 medium supplemented with rhamnose (0.2%), histidine, and tryptophan. In cases where evolved alleles contained multiple mutations, overlapping PCR fragments derived from mutant and wild-type templates were used to reconstruct specific alleles. Deletions, insertions, and selected point mutations were constructed using DIRex with half-cassettes derived from *Atox1* and *Atox2* (GenBank MN207490), as described previously (Näsvall 2017; Näsvall 2022). All oligonucleotides are listed in Supplementary Table 2.

### Generation of a mutator allele

A reversible mutator allele was constructed by insertion of the selectable and counter-selectable Acatsac1 cassette (GenBank accession MF124798) into mutS using a duplication–insertion (DUP-In) strategy (Näsvall et al. 2017; Näsvall 2022). The insertion generated 2 truncated copies of *mutS*, each lacking essential parts of the *mutS* coding sequence, separated by the *Acatsac1* cassette. As neither truncated copy is expected to encode a functional MutS protein, the resulting strains displayed a mutator phenotype. The DUP-In design allows reversion of the mutator allele by recombination between duplicated sequences and loss of the cassette upon selection on sucrose, although reversion was not tested in this study.

### Constitutive expression of *trpA* and *trpF* from a heterologous locus

To assess the effects of *trpA* mutations on TrpA and TrpF functions separately, wild-type *trpA* and *trpF* genes were expressed from the strong constitutive *P*_CP25_ promoter (Jensen and Hammer 1998) at a chromosomal locus unrelated to the native tryptophan biosynthetic operon. Recipient strains carried a *P*_CP25_-driven Red fluorescent protein (*mScarlet*) gene inserted at 1 of the 6 IS*200* elements in the *S. enterica* genome, along with deletions of either *trpA* or *trpF*. λ Red recombineering was used to replace the mScarlet coding sequence with *trpA* or *trpF*, selecting for growth in the absence of tryptophan. To facilitate transfer of the *P*_CP25_-*trpA* and *P*_CP25_-*trpF* constructs to other strains, a duplication containing a selectable and counter-selectable marker (*dhfr*–*P_rhaB_*–*orph11*) was inserted adjacent to the same locus. Following generalized transduction and selection for trimethoprim resistance, loss of the marker was selected by growth in the presence of rhamnose, yielding marker-free strains. Initial tests showed that *P*_CP25_-*trpA* fully complemented a Δ*trpA* (*trpF*^+^) strain but failed to restore growth of reconstructed *trpA ΔtrpF*) mutants. Because TrpA functions in concert with TrpB as an α_2_β_2_ complex (Pittard and Yang 2008), competition between wild-type and mutant TrpA proteins for TrpB binding may inhibit the function of the mutant TrpA variants. To address this, a *P*_CP25_-*trpBA* construct co-expressing *trpB* and *trpA* was generated.

### Phage P22 methods and construction of Gene Transfer Agent GTA22

Generalized transduction between *Salmonella enterica* strains was primarily performed using phage P22 HT105/1 *int-201* (Schmieger 1972). In long-term evolution experiments and repeated transductions, contamination by P22 or selection for P22-resistant mutants can complicate downstream genetic analyses. To minimize these issues, 2 complementary strategies were used.

In evolution experiment 1, strains carried deletions in the *gal* operon (Δ*P_gal_* Δ*galE*::*T_lux_*). GalE catalyzes the reversible interconversion of UDP-galactose and UDP-glucose, and loss of GalE results in conditional truncation of the O-antigen in the absence of galactose. Because phage P22 uses the O-antigen as a receptor, this modification confers resistance to P22 infection in the absence of galactose while maintaining sensitivity in its presence. To avoid galactose sensitivity due to toxic accumulation of UDP-galactose, the *gal* operon promoter was deleted and a transcriptional terminator was inserted in place of *galE*, reducing expression of *galK* and *galT*.

To facilitate repeated transductions while preventing lytic growth of phage P22, an inducible artificial gene transfer agent (GTA22) was constructed using λ Red recombineering. GTA22 was derived from a P22 HT105/1 *sieA44* lysogen by 3 major genetic modifications. First, both ends of the prophage, including the attachment sites, were deleted using DIRex (Näsvall 2017; Näsvall 2022). One deletion Δ*thrW*–*kil*) removed approximately 6.9 kb from the left end, including the integrase and excisionase genes (*int* and *xis*), recombination genes (*abc1*, *abc2*, *erf*), and the septation inhibitor gene *kil*. A second deletion removed approximately 3 kb from the right end, including the attachment site duplicate and the O-antigen conversion locus *gtrABC*. Second, a copy of the P22 antirepressor gene (*ant_P22_*) was placed under the control of the arabinose-inducible *P_araBAD_* promoter. Because packaging initiates at a single *pac* site and proceeds unidirectionally (Susskind and Botstein 1978), packaging of a complete GTA22 genome into a single virion, resulting in a virulent phage particle, is expected to be rare. To induce GTA22, overnight cultures were diluted 1:20 into LB supplemented with 0.1% (w/v) L-arabinose and incubated for at least 6 h at 37 °C to allow lysis. Surviving bacteria were killed with chloroform. Lysates were clarified by centrifugation (≥14,000 × g for 1 to 3 min) and used directly in transductions. For transduction, 0.1 to 10 μL of cleared lysate was mixed with 100 μL LB supplemented with 0.4% glucose to repress *P_araBAD_* in recipient cells, followed by the addition of 100 μL recipient overnight culture. After 30 min incubation at 37 °C, mixtures were plated on selective media.

### PCR, sequencing, and bioinformatic analysis

#### PCR and Sanger sequencing

PCR products for λ Red recombineering and Sanger sequencing were generated using Phusion DNA polymerase (Thermo Fisher). PCR templates consisted of bacterial cell suspensions prepared either from a portion of a single colony or from ∼1 μL of frozen stock culture resuspended in 100 μL ultrapure water. One microliter of suspension was used per 20 μL PCR reaction. Cycling conditions have been described previously (Näsvall 2017; Näsvall et al. 2017; Näsvall 2022). PCR products were purified by PEG precipitation using a 2× solution containing 24% (w/v) PEG 8,000 and 20 mM MgCl_2_. DNA pellets were washed with 70% ethanol, air-dried, and resuspended in ultrapure water. For Sanger sequencing, purified PCR products were mixed with sequencing primers (Supplementary Table 2) and submitted to Eurofins Genomics (Ebersberg, Germany).

#### Whole-genome re-sequencing (WGS)

Genomic DNA was extracted from evolving populations using MasterPure DNA purification kits (Epicenter). Libraries for MiSeq sequencing (in-house) were prepared using Nextera XT DNA library preparation and indexing kits (Illumina). Some samples were sequenced by BGI (Beijing, China) using DNBseq technology. Reads were processed and analyzed using CLC Genomics Workbench. Reads were trimmed (quality limit: 0.001) and mapped (length fraction: 0.9, similarity fraction: 0.9) to a reference genome containing all known modifications in the ancestral strain. Read mappings were realigned using the Local Realignment tool, after which mutations were called using CLCs Low Frequency Variant Detection tool (1% significance). InDels and rearrangements were detected using the InDels and Structural Variants tools. Potential rearrangements (insertions, inversions, deletions, and duplications between short repeats) reported by the Structural Variants tool were further analyzed by comparing the nonmapping part of partially mapped reads to other parts of the reference genome. This resulted in identification of one duplication present in one population (between *phsA* and *wzzB*, including the entire *his* operon), 2 inversions (in the phase variable systems *hin*, present in most populations, and *fin*, present in one population), one deletion (a 98 bp deletion in the *trpE* gene present in one population), but no transposition events (Supplementary Tables 3–6). Duplications between long repeats (that cannot be detected as partially mapped reads) were found by visual scanning of the mapped read depth, resulting in the identification of 2 large duplications between *rrn* operons. Summaries of WGS analysis, including identified mutations and their frequencies, and mean coverage for the chromosome and the pSLT virulence plasmid for all sequenced strains, are listed in Supplementary Tables 3–6. Copy number estimates for the region amplified in DA65458 were derived from mapped read depth using the QC for targeted sequencing tool in CLC (Supplementary Table 7), comparing the read depth of the amplified region with 4 nearby regions of identical size.

#### Isolation and sequencing of clones from evolved populations

To determine the order of appearance of mutations in populations that accumulated multiple mutations in *hisA* or *trpA*, clones were isolated from frozen population samples taken at earlier time points. Frozen stocks were streaked on LA plates, and 8 single colonies per population were restreaked once more for purification. From each purified clone, a single colony was used as a template for PCR amplification of the target gene, followed by Sanger sequencing (Supplementary Table 8).

### Experimental evolution

Two experimental evolution studies were performed (outlined in Fig. 1).

**Experiment 1** was initiated from 8 independent cultures, started from separate colonies, of strain DA52864 (Δ*trpF* Δ*Pgal* Δ*galE*::*T_lux_*). Following overnight growth in medium supplemented with histidine and tryptophan, cultures were diluted 1:100 into 5 mL of tryptophan-limited medium (2× M9 + 5 μM tryptophan) and propagated by serial transfer; each 1:100 dilution resulted in approximately 6.6 (log_2_ 100) population doublings per cycle. The tryptophan concentration of 5 µM was chosen as it allows the Δ*trpF* ancestor to grow at a rate indistinguishable from the wild-type but limits the final density to an *OD*_600_ of approximately 0.2 (Supplementary Figs. 2–5). This concentration, therefore, was thought to provide a sufficient population size in 5 mL cultures to ensure mutational target availability while leaving significant room for improved final yield upon the acquisition of tryptophan prototrophy. Cultures were passaged once every 7 d for 20 cycles and subsequently twice weekly with 1:1000 dilutions for an additional 30 cycles, totaling approximately 430 population doublings. The 7-day cycling protocol was chosen to allow enrichment of slow-growing, growth-rescuing mutants under conditions where the ancestor could not grow. Samples were frozen at −80 °C in 20% DMSO at multiple time points.

**Experiment 2** was initiated from strains DA62207 (Δ*trpF*) and DA64168 (Δ*trpF mutS*), each carrying GTA22 (described above). Forty-eight independent populations, started from separate colonies, were passaged weekly by 1:100 dilution in tryptophan-limited medium for 13 cycles. Samples were frozen at −80 °C in 20% glycerol at multiple time points.

### Phenotypic characterization and growth kinetics

#### Growth assays

Growth assays (Figs. 2–4, Supplementary Figs. 2–6, 8 and 9) were initiated from overnight cultures grown in 2× M9 supplemented with glucose, histidine, and tryptophan. Additionally, due to an unexpected negative effect of tryptophan addition to the growth of *hisA* mutants, we supplemented the medium with guanosine when needed (See Supplementary Results and Discussion). Cultures were diluted 1:1000 into tryptophan-free or tryptophan-limited medium and transferred to Bioscreen C honeycomb plates. OD600 was recorded every 4 min for up to 7 d at 37 °C with continuous shaking. During assays, evaporation from wells at the edges of the plates was occasionally observed despite sealing. In early experiments, edge wells sometimes reached higher final OD values and showed visible volume loss. However, up to approximately 90% of maximum OD, evaporation did not appear to substantially affect growth measurements. In later experiments, edge wells were filled with medium but not used for cultures to minimize evaporation effects. For fitness comparisons based on AUC, analyses were restricted to the first 72 h. Some strains displayed fluctuations in OD600 following depletion of tryptophan, interpreted as entry into the death phase followed by partial regrowth due to recycling of nutrients. Complementation assays using *P*_CP25_ constructs (described above) evaluated the restoration of function in mutant backgrounds (Supplementary Figs. 8 and 9).

### Growth curve analysis and kinetic modeling

Bacterial growth kinetics were analyzed using custom R scripts. Growth profiles in tryptophan-limited medium were partitioned into 2 distinct phases for analysis: predepletion (early exponential phase) and postdepletion (secondary growth phase). A summary of the growth metrics and statistics derived from the growth curves can be found in Supplementary Table 9.

Growth constants (*k*_pre_) were determined by fitting raw *OD*_600_ data to an exponential model: *Y(t)* = *b* + *Y_0_* • *e^kt^*, via the minpack.lm package. The algorithm initially considered all data points where *OD*_600_ < 0.3; if the fit quality did not meet *R*^2^ ≥ 0.999, the latest time points were iteratively removed. Secondary growth kinetics were characterized for the interval following predepletion. The start of this phase was detected by calculating rolling slopes over a 20-point window to identify when the local growth rate dropped below 85% of the initial *k*_pre_ rate. The interval ended when the culture reached 90% (evolving populations in Fig. 2) or 93% (reconstructed and complemented strains in Fig. 3) of its measured maximum optical density *OD*_max_. Total growth was quantified as the Area Under the Curve (AUC) over 72 h using the trapezoidal rule.

### Statistical analysis

A priori statistical comparisons were performed to evaluate differences in growth kinetics between mutants and their respective ancestors. Significant differences were assessed using two-tailed, unpaired Welch's *t*-tests (*n* = 4 or 5 biological replicates). To control the false discovery rate, *P*-values were adjusted using the Benjamini–Hochberg (BH) method. Significance levels are reported as * *P* < 0.05, ** *P* < 0.01, and *** *P* < 0.001. All calculations and visualizations were performed in R (v4.5.2) using the minpack.lm, ggplot2, and igraph packages.

## Results

### Experimental evolution restores growth of Δ*trpF* strains under intermittent selection

To identify genetic changes that enable growth of *Salmonella enterica* strains lacking *trpF* under minimally biased conditions, we evolved replicate populations under intermittent selection for rescue of tryptophan prototrophy. Because loss of *trpF* prevents growth only after depletion of exogenous tryptophan, cultures were propagated in medium containing a limiting amount of tryptophan (5 µM). This specific concentration was selected to support an initial population size large enough to sustain the lineages while ensuring that the ancestor entered the stationary phase at a low density, thereby providing a clear selective advantage to any mutants capable of independent tryptophan synthesis (Fig. 1).

**Fig. 1.**
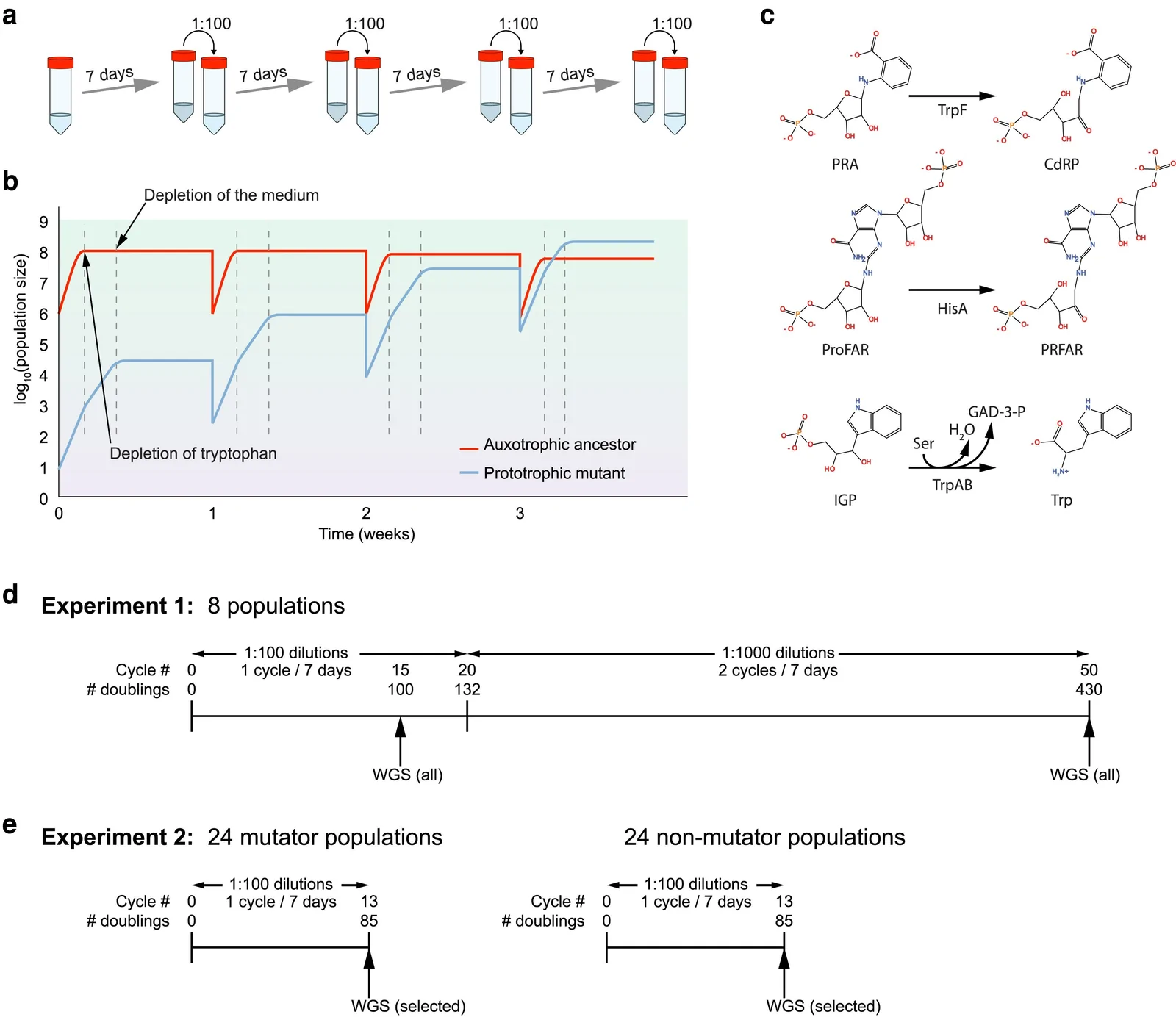
Experimental outline. a) Serial passage experiments with *Salmonella enterica* lacking the *trpF* gene, grown in media with limiting tryptophan (5 µM). Each 7-day cycle consisted of a short period with unlimited growth (tryptophan present) followed by a longer period without tryptophan (after tryptophan has been depleted). Only prototrophic mutants (that had evolved to rescue the growth of the Δ*trpF* mutant in the absence of tryptophan) could grow during this phase. b) The expected dynamics during the takeover of a batch culture population by a growth-rescuing mutant competing with its Δ*trpF* ancestor in tryptophan-limited medium. Each cycle includes a brief window where the growth-rescuing mutant can grow and outcompete the ancestor. The ancestor is expected to cease growing when tryptophan is depleted while the growth rescuing mutant continues growing until the medium is depleted for some other essential nutrient(s). c) Reactions catalyzed by TrpF, HisA, and the TrpAB heterotetrameric complex. See Supplementary Fig. 1 for the complete trp pathway and the reaction mechanisms of TrpF, HisA, and TrpAB. (d, e) Evolution timelines for experiments 1 and 2, respectively, with line lengths scaled to population doublings. Number of passages (cycle #), dilution factors, and cumulative population doublings are indicated above the lines. Vertical arrows denote timepoints sampled for whole-genome sequencing (WGS).

In experiment 1, 8 populations were evolved for approximately 430 generations. By the end of the experiment, 3 populations (1-1, 1-3, 1-7) exhibited detectable growth after tryptophan depletion (Fig. 2a; Table 1). Whole-genome sequencing (WGS) revealed that populations 1-1- and 1-3 carried multiple amino acid substitutions in *hisA* and harbored mutator mutations in *mutH* or *mutS* (Table 1 and Supplementary Table 3). WGS of the same populations after approximately 100 generations revealed that several mutations, including the mutator alleles, that were present at high frequency in the end-point populations were detectable at low frequencies at this early time point before any of the *hisA* mutations were detectable (Supplementary Table 4). Population 1-7 achieved growth rescue without mutations in *hisA* or *trpA*, leaving its genetic basis unresolved.

**Fig. 2.**
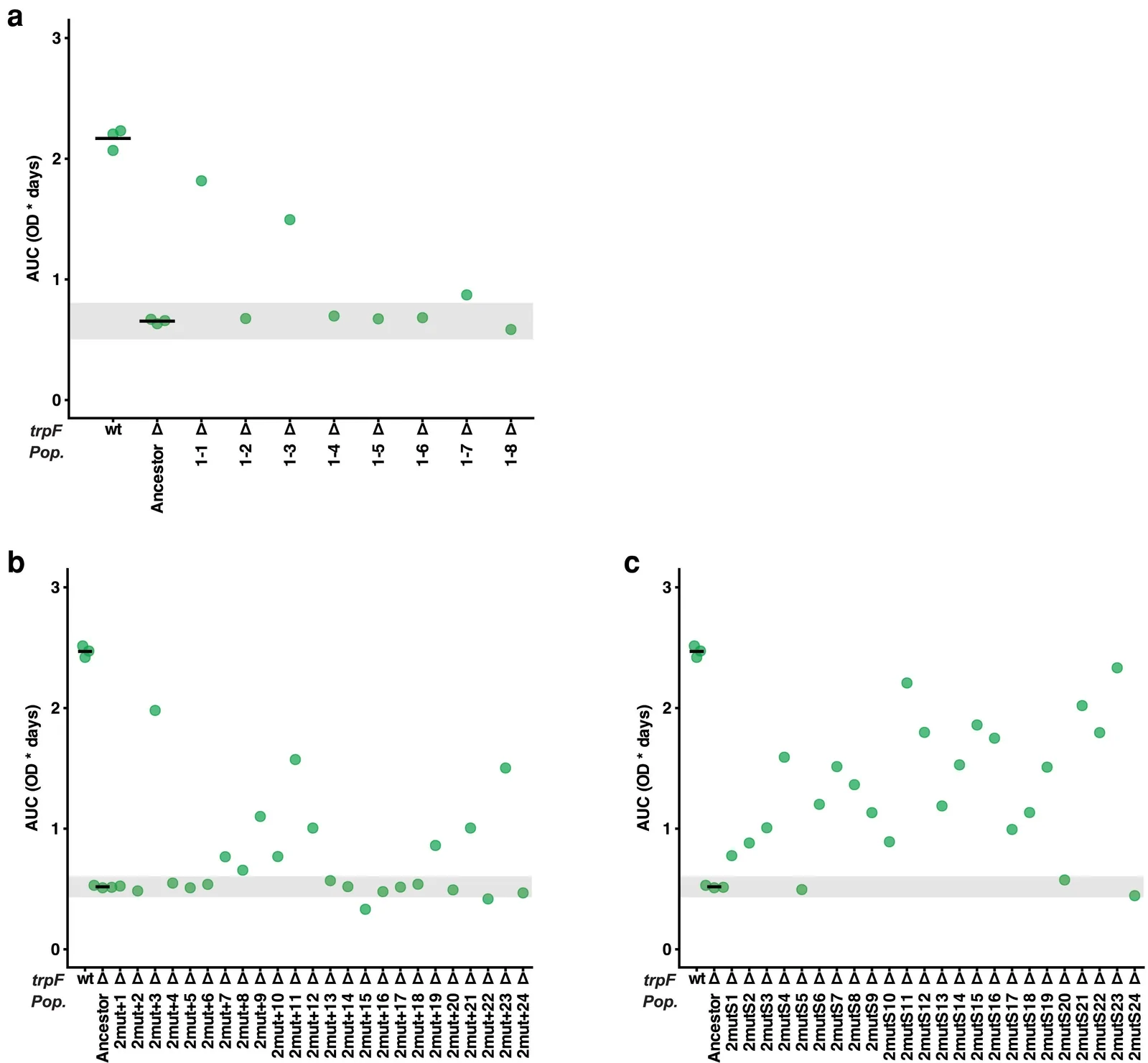
Growth of evolved populations in tryptophan-limited medium. a) The 8 populations from experiment 1. b) The 24 nonmutator populations from experiment 2. c) The 24 mutator populations from experiment 2. Growth is reported as the area under the curve (AUC) of OD_600_ vs time for the first 3 d of growth. The *trpF*+ (wt) and the Δ*trpF* (ancestor) strains were grown as 3 biological replicates, and the populations were grown from a portion of the freezer stocks of the end-point populations. The black lines (wt and ancestor) represent the mean of the replicates. All populations that were considered to have evolved a Δ*trpF* growth rescue function have AUCs above the 98% prediction interval (grey shaded areas) of the ancestor. See Supplementary Fig. 5 for growth curves from the same experiment.

**Table 1.** Identified mutations conferring Δ*trpF* growth rescue activity.

Strain #	Population^a^	# Generations^b^	Causative mutation(s)^c^	Identified by^d^
DA59062	1-1	430	*hisA*[Q18R A22T R23L K107E V168A]	WGS
DA59064	1-3	430	*hisA*[Q18R A22T V168A]	WGS
DA59068	1-7	430	(not identified)^e^	WGS
DA65429	2mut + 3	85	*trpA*[P62fs]	WGS
DA65433	2mut + 7	85	*trpA*[P62fs]	WGS
DA65434	2mut + 8	85	*trpA*[P62fs]	WGS
DA65435	2mut + 9	85	*trpA*[P62fs]	WGS
DA65436	2mut + 10	85	*trpA*[P62fs]	WGS
DA65437	2mut + 11	85	*trpA*[P62fs]	WGS
DA65438	2mut + 12	85	*trpA*[P62fs]	WGS
DA65445	2mut + 19	85	*trpA*[P62fs]	WGS
DA65447	2mut + 21	85	*trpA*[P62fs], *trpA*[A67T]	WGS
DA65449	2mut + 23	85	*trpA*[P62fs]	WGS
DA65451	2mutS1	85	*hisA*[Q18R]	Sanger
DA65452	2mutS2	85	*hisA*[Q18R A127G]	WGS
DA65453	2mutS3	85	*hisA*[Q18R H17R]	WGS
DA65454	2mutS4	85	*trpA*[D27Y G98S]	Sanger
DA65456	2mutS6	85	*trpA*[P62fs]	Sanger
DA65457	2mutS7	85	*trpA*[P62fs]	Sanger
DA65458	2mutS8	85	*hisA*[Q18R A22T] (8-fold amplified)^f^	WGS
DA65459	2mutS9	85	*hisA*[Q18R]	Sanger
DA65460	2mutS10	85	*hisA*[Q18R]	Sanger
DA65461	2mutS11	85	*trpA*[G98C]	Sanger
DA65462	2mutS12	85	*trpA*[P62fs]	WGS
DA65463	2mutS13	85	*trpA*[P62fs]	Sanger
DA65464	2mutS14	85	*trpA*[G98S Y102H Q250R]	Sanger
DA65465	2mutS15	85	*trpA*[P62fs]	Sanger
DA65466	2mutS16	85	*trpA*[T24S G61S P62S]	WGS
DA65467	2mutS17	85	*hisA*[Q18R]	Sanger
DA65468	2mutS18	85	*hisA*[Q18R]	WGS
DA65469	2mutS19	85	*hisA*[Q18R T52S]	Sanger
DA65471	2mutS21	85	*trpA*[P62fs]	Sanger
DA65472	2mutS22	85	(not identified)^e^	WGS
DA65473	2mutS23	85	*trpA*[D27Y G98C]	WGS

^a^Only populations that could grow more turbid than their ancestor within 7 d at the end of the experiment are listed. The populations are named according to which evolution experiment (1 or 2) they were part of, and the order the cultures were handled in during passages (1 to 8 and 1 to 24, respectively for the first and second evolution experiment). The populations from the second experiment additionally have “mut+” for nonmutators and “*mutS*” for mutators.

^b^The numbers of generations were calculated from the fold dilution and the number of passages [log_2_(dilution)×passages] and reflect the total number of population doublings.

^c^The mutations listed have been re-constructed in the genetic background of the nonevolved ancestral strain (Δ*trpF*), and has been verified to confer growth in the absence of tryptophan.

^d^Mutations were identified either through whole genome sequencing (WGS) or Sanger sequencing (targeting the *trpA* and *hisA* genes). For a complete list of mutations present at high frequencies in the whole-genome sequenced populations, see Supplementary Tables 3–6.

^e^These populations did not have any mutations in *hisA* or *trpA*.

^f^This population contained a ∼19 kb amplification encompassing the entire *his* operon at ∼8-fold coverage compared to the surrounding genomic DNA.

In Experiment 2, 48 populations (24 *mutS* mutators and 24 nonmutators) were evolved for 85 population doublings. Growth rescue was observed in 21 mutator and 10 nonmutator populations (Fig. 2b and c). Candidate mutations sufficient for the phenotype were identified in *hisA* (9 populations) or *trpA* (21 populations; Table 1), leaving one population (2*mutS*22) lacking mutations in *hisA* or *trpA* unresolved.

### The *trpA*(P62fs) frameshift is a frequent and sufficient solution

A 7-bp duplication in *trpA,* causing a frameshift (P62fs), was identified in all 10 nonmutator populations and 6 mutator populations from experiment 2. While cross-contamination between populations cannot be entirely ruled out, the mutation is highly likely to have arisen through independent, parallel evolution, as the WGS analysis revealed no other mutations that exclusively co-occurred with P62fs (Supplementary Tables 5 and 6).

To investigate the mechanisms underlying this widespread rescue, we first sought to determine if this mutation required specific background adaptations. We reconstructed the P62fs allele in an otherwise unevolved Δ*trpF* genetic background. The reconstructed P62fs allele was sufficient to confer growth in the absence of tryptophan (Fig. 3b, Supplementary Fig. 4, a and b), confirming that this single mutation was the primary driver of the rescue phenotype. However, because this mutation occurred early in the gene and resulted in a premature stop codon, the resulting 83-amino acid peptide from the original reading frame was unlikely to be functional, necessitating frameshift suppression to explain the phenotype. We tested a specific hypothesis—a + 1 translational frameshift at a downstream proline (CCC) codon—by constructing a nonframeshifted variant (*trpA*[61-62insRW]; Supplementary Fig. 7) that encodes the protein product predicted by this suppression mechanism. While this “suppression-mimic” mutant was capable of supporting growth, it did so to a lesser degree than the original P62fs mutant (Supplementary Figs. 8 and 9). This suggests that while the proposed frameshifting mechanism may contribute to the phenotype, it is insufficient to explain the full extent of the growth rescue observed in the P62fs mutant.

### Mutator status shifts the mutational spectrum toward SNP-based solutions

While *hisA*-based solutions were found only in mutator backgrounds, mutator status appeared to facilitate access to these routes by altering the global mutational spectrum. Across all sequenced mutator populations, we observed a strong transition bias: 74% of mutations were transitions compared to 11% transversions (Supplementary Tables 3 and 5). In nonmutators, this bias was notably lower (38% transitions vs 27% transversions; Supplementary Tables 3 and 5).

This shift is reflected in the target genes: in nonmutator populations, the adaptive landscape was dominated by the *trpA*(P62fs) duplication (91% of target mutations; Supplementary Table 5), with only a single SNP identified. In contrast, mutator populations frequently accessed point mutations (Supplementary Tables 3 and 6); transitions accounted for 72% of mutations in *hisA* and *trpA*. Every *hisA*-based path began with the same A→G transition (Q18R; Supplementary Tables 3, 6 and 8). As for the *trpA*[P62fs] mutation, the lack of co-occurring potential passenger mutations shared exclusively between populations with the Q18R mutation argues for its independent formation in multiple lineages (Supplementary Table 6). These data suggest that elevated SNP frequencies in mutators allow populations to sample a wider array of amino acid substitutions, shifting the bias away from the frequent *trpA*(P62fs) duplication.

### Gene duplication is rare and does not lead to detectable functional divergence

In the minimal medium used for evolution, growth requires that both histidine and tryptophan biosynthesis remain functional. Despite previous models suggesting that gene duplication is a primary route to new functions, only one whole-genome sequenced population (2*mutS*8) harbored an amplification of a target gene. This population carried an approximately 8-fold amplification of the *his* operon encompassing the *hisA*(Q18R A22T) allele (Supplementary Table 7). Sequencing reads showed 100% agreement with this mutant allele, with no evidence for the maintenance of a wild-type copy or further divergence between the copies (Supplementary Table 6). In all other populations, adaptation occurred exclusively through point mutations or small indels in single-copy genes.

### Mutational paths are highly constrained in *hisA* but diverse in *trpA*

To determine the order of mutation accumulation, we sequenced clones from intermediate time points (Supplementary Table 8). All evolutionary paths involving *hisA* began with the same amino acid substitution, Q18R. This mutation appears to be a prerequisite for further adaptation in this gene, consistent with the observation that single substitutions in *hisA* rarely enable growth without severe tradeoffs. In contrast, *trpA* exhibited greater mutational diversity; initial mutations included G98C, D27Y, and Y102H, suggesting a broader range of accessible paths for this locus.

### Successive mutations improve growth rescue under selection

We reconstructed 23 different *hisA* and *trpA* variants in an unevolved background to isolate their effects. All reconstructed variants enabled growth in the absence of tryptophan (Supplementary Figs. 3–5). We reconstructed strains representing every mutation identified throughout the experiment. In 2 instances where the order of specific mutations (T24S and G61S, and G98C and D27Y in *trpA*) could not be resolved, we generated both possible intermediate genotypes. In most mutational pathways, each additional mutation resulted in a statistically significant improvement in growth (Fig. 3). While all *hisA* and *trpA* variants were sufficient to restore tryptophan prototrophy (Supplementary Figs. 3–5), it is important to note that our primary measure of “function” is physiological. While the acquisition of TrpF-like catalytic activity is the most parsimonious explanation for this growth rescue, consistent with prior biochemical studies of HisA and TrpA, our experiments demonstrate the sufficiency of these alleles for the growth phenotype rather than providing direct measurements of enzyme kinetics or protein-level bifunctionality. Consequently, these results characterize the genetic basis of adaptation under our selective regime without excluding potential alternative physiological mechanisms for tryptophan prototrophy.

**Fig. 3.**
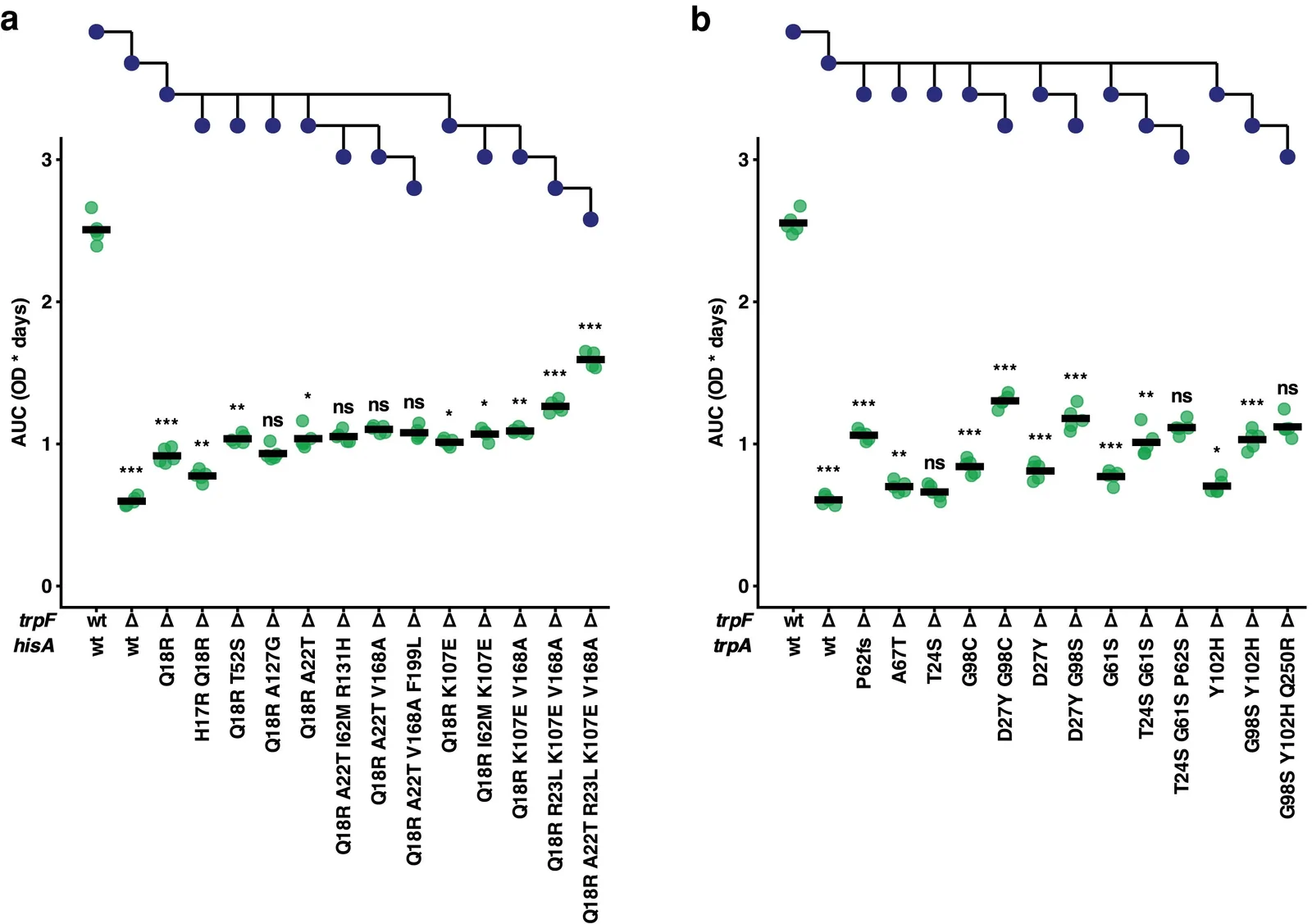
Growth of re-constructed mutants in tryptophan-limited medium. a) *hisA* mutants. b) *trpA* mutants. Reconstructed mutants were grown in M9 glucose minimal medium supplemented with limiting tryptophan (5 μM) and guanosine (0.3 mM; see Supplementary Results and Discussion). Growth is reported as the area under the curve (AUC) for the first 3 d. Black bars represent averages. The lineage maps above the plots indicate the order of appearance of each mutation as deduced from sequencing of isolated clones from various time-points in the evolving populations. ns, nonsignificant (*P* > 0.05); *, *P* < 0.05; **, *P* < 0.01; ***, *P* < 0.001, based on a Welch's *t*-test, corrected for multiple testing using the Benjamini–Hochberg method. Each mutant was compared to its immediate ancestor, as indicated in the lineage maps. All strains were grown in at least 4 independent biological replicates. Only the 2 leftmost *trpA* mutants (P62fs and A67T) were found in nonmutator populations, all others (in both genes) were found in only hypermutators. The growth curves can be found in Supplementary Figs. 3–5.

### Trade-offs with ancestral function are more severe in *hisA* than in *trpA*

To measure tradeoffs, we assessed the growth of reconstructed mutants under conditions requiring their ancestral functions.

**HisA tradeoffs:** All reconstructed *hisA* mutants grew significantly slower than the wild-type strain prior to tryptophan depletion (Fig. 4a, Supplementary Figs. 3 and 4). Successive mutations that improved tryptophan rescue further decreased the growth rate in histidine-requiring conditions, reflecting a decline in native HisA function or expression.

**Fig. 4.**
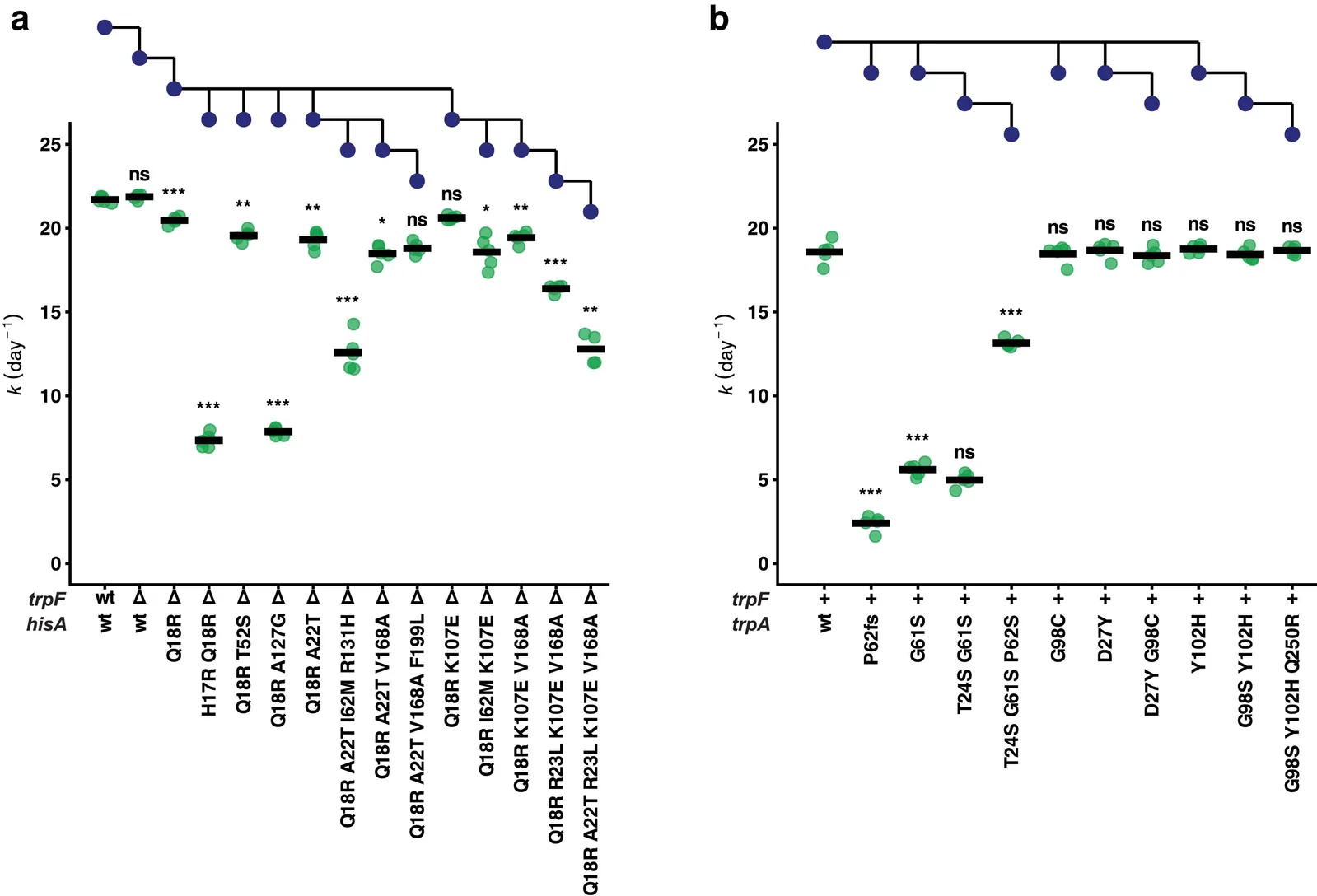
Impact of mutations on original function evolution of Δ*trpF* growth rescue. a) Growth dependent on *hisA* function: Exponential growth rates were measured in the early, rapid growth phase before tryptophan depletion in M9 glucose medium supplemented with limiting tryptophan (5 μM) and guanosine (0.3 mM; See Supplementary Results and Discussion). Rates were normalized to the growth rate of the *trpF*+ wild-type strain grown in the same experiment. Under these conditions, reductions in *hisA* function lead to decreased growth rates. See Supplementary Figs. 3 and 4 for growth curves. b) Growth dependent on *trpA* function: Exponential growth rates were measured in M9 glucose medium without supplements for strains expressing wild-type *trpF* from a constitutive promoter at a different locus. Here, reductions in *trpA* function lead to decreased growth rates. For *trpA* mutants under the same conditions as (a), see Supplementary Fig. 6. Black bars represent averages. The lineage maps above the plots indicate the order of appearance of each mutation as deduced from sequencing of isolated clones from various time-points in the evolving populations. ns, nonsignificant (*P* > 0.05); *, *P* < 0.05; **, *P* < 0.01; ***, *P* < 0.001, based on a Welch's *t*-test, corrected for multiple testing using the Benjamini–Hochberg method. Each mutant was compared to its immediate ancestor, as indicated in the lineage maps. All strains were grown in at least 4 independent biological replicates.

**TrpA tradeoffs:** All *trpA* mutants grew at wild-type rates in the presence of exogenous tryptophan (Supplementary Figs. 5 and 6). To specifically test for TrpA-dependent growth, we provided a wild-type *trpF* gene in trans. Under these conditions, 6 out of 10 *trpA* mutants were indistinguishable from the wild-type, indicating they retained full ancestral function. Four mutants showed reduced growth, which was partially restored by supplying *trpA* and *trpB* in *trans*, suggesting that some alleles do incur tradeoffs with native TrpA function or expression (Fig. 4b, Supplementary Figs. 8 and 9).

### Unidentified mechanisms of trp-independent growth

In 2 populations (1-7 and 2*mutS*22), we identified growth rescue that did not involve mutations in *hisA* or *trpA*. In population 2*mutS*22, the phenotype could not be transferred to the ancestral strain via single-gene transduction. This suggests a more complex, potentially polygenic basis for adaptation in these specific lineages.

## Discussion

### Mutational routes to rescue of *trpF* deletion

By evolving Δ*trpF* populations under a selective regime designed to reduce experimental bias, we found that point mutations in multiple genes (*hisA* and *trpA*) can repeatedly rescue growth without exogenous tryptophan while retaining sufficient ancestral growth-supporting functions. However, these were not the only solutions accessible under our regime; in 2 populations, growth rescue occurred through as-yet unresolved mechanisms that did not involve mutations in *hisA* or *trpA*. In at least one of these cases, the phenotype was not transferable as a single-gene change, suggesting more complex or polygenic adaptations. While point mutations in *hisA* and *trpA* represent the most frequent and accessible single-gene routes, these unresolved cases highlight the existence of alternative evolutionary paths to tryptophan prototrophy.

Our results show that the growth of Δ*trpF* strains under intermittent selection can be restored through mutations in multiple genes without the necessity of gene duplication and divergence. Mutations in *hisA* and *trpA* both supported rescue of tryptophan prototrophy but differed in their mutational accessibility and in the extent to which ancestral growth-supporting functions were compromised. Specifically, *hisA*-based solutions were associated with stronger tradeoffs and were largely facilitated by mutator backgrounds, whereas *trpA*-based solutions were more frequent and often retained full native function.

Several of the mutations identified in our study have been observed in previous directed evolution studies. In *hisA*, the Q18R and A127G substitutions were previously found in a selection for restored tryptophan prototrophy, and represent 2 of only 3 known single amino acid substitutions in *hisA* mutations that rescue growth of a Δ*trpF* strain without complete loss of native HisA function. The remaining *hisA* mutations found in our experiments—H17R, T52S, A22T, I62M, R131H, F199L, K107E, and R23L—were isolated during rounds of mutagenesis and selection for improved Δ*trpF* growth rescue, but they always occurred in combination with other mutations and their individual phenotypic effects were not tested in isolation (Lundin et al. 2020).

In *trpA*, the mutations G61S, P62S, and G98C, as well as a substitution at position 24 (T24A), were previously observed by Evran et al. (2012). Based on their results, these authors concluded that these mutations were not sufficient on their own, but instead either permitted or enhanced TrpF activity (confirmed by enzyme kinetics) in a TrpA variant engineered to contain the catalytic residues from TrpF. However, the effects of the individual mutations were not tested, and the necessity of the introduced catalytic residues was not confirmed. In light of our present results, where these mutations are sufficient for Δ*trpF* growth rescue without additional *trpA* mutations, the conclusions of Evran et al. may need to be revised (see Supplementary Results and Discussion).

While these comparisons demonstrate that many of the identified mutations can be accessed across different experimental regimes, the consistent emergence of bifunctional genetic solutions is directly linked to our selective conditions.

### Intermittent selection promotes gene-level generalism

This study was designed to identify genetic solutions that restore growth of Δ*trpF* bacteria under conditions intended to minimize experimental bias. A central result is that Δ*trpF* growth rescue emerged via mutations in at least 2 different genes, *hisA* and *trpA*, across independent populations. Unlike previous experimental designs that relied on continuous strong selection or engineered alleles (Evran et al. 2012; Näsvall et al. 2012; Lundin et al. 2020), our framework utilized intermittent selection via nutrient depletion.

Fluctuating selection is known to promote generalist phenotypes at the organismal level, particularly when adaptation involves tradeoffs across environments (Kassen 2002; Legros and Koella 2010; Hall et al. 2013; Arribas et al. 2014; Kortright et al. 2022; Fasanello et al. 2024). Our results suggest that a similar logic applies at the gene level: when selection alternates between phases emphasizing different requirements, evolution may favor alleles that satisfy multiple requirements simultaneously, even if those alleles are not optimal for either requirement alone. Under the cycle structure used here, a growth-rescue mutation must enable sufficient postdepletion growth to compensate for any predepletion deficit before the medium becomes exhausted.

### Divergence from the innovation-amplification-divergence (IAD) model

A notable difference from prior work is the low prevalence of gene duplication or amplification. In earlier designs using amplification-prone genomic contexts and constitutive expression, duplication and subsequent divergence were central features (Näsvall et al. 2012). However, in this study, only one population showed evidence of amplification, and it lacked divergent alleles.

Several aspects of the present design likely contributed to this outcome, including the use of native chromosomal contexts and regulation. While we cannot exclude transient duplications, their absence in the majority of end-point populations suggests that duplication was not a dominant route. This is consistent with the expectation that many duplications carry fitness costs and require sustained positive selection to be maintained (Hill et al. 1969; Haack and Roth 1995; Reams et al. 2010). Under intermittent selection, any benefit of duplication restricted to the postdepletion phase could be reduced by loss during phases lacking selection for growth rescue.

### Mutational accessibility and bifunctionality through exceptions from standard translation

The repeated appearance of the *trpA*(P62fs) frameshift mutation in experiment 2 indicates it is a highly accessible and competitive route to Δ*trpF* growth rescue. As the truncated 83-amino acid peptide resulting from this frameshift is highly unlikely to be functional, we hypothesize that this mutant relies on efficient frameshift suppression to support growth. This suppression likely allows translation to yield a full-length protein with an altered amino acid sequence that retains enough native activity to support growth while also providing Δ*trpF* growth rescue. We tested a hypothesis for how this growth rescue could be achieved, and found that while a gene that encoded the protein that would result from that mechanism did support Δ*trpF* growth rescue and still retained the *trpA* function, the tested mechanism could not explain the full extent of the observed growth rescue (Supplementary Figs. 7–9).

The identification of mutations in ribosomal components (e.g. RsmD, S2, and L9) in these populations provides a link to translational suppression mechanisms (Leipuviene and Björk 2007; Arora et al. 2013). This suggests that the evolutionary path to bifunctionality can leverage exceptions from standard translation, allowing a single genetic sequence to produce the full-length polypeptide(s) required to satisfy both physiological requirements simultaneously. Similarly, in our previous experiments (Näsvall et al. 2012; Newton et al. 2017), we identified a single amino acid substitution in *hisA* (L169R, CUG→CGG) that generated TrpF activity but lost all detectable native activity. An extragenic suppressor of this mutation altered the anticodon of a tRNA^Gln^ (from CUG to CCG), allowing it to insert glutamine on CGG (arginine) codons. A constructed *hisA*(L169Q) mutant grew in conditions requiring the histidine pathway but not in conditions requiring the tryptophan pathway. Strains with the *hisA*(L169R) mutation and the missense suppressor tRNA thus made 2 relevant polypeptides from a single *hisA* gene, essentially making the *hisA* gene bifunctional by allowing the expression of 2 separate polypeptides through a translational exception.

### Physiological bifunctionality vs biochemical efficiency

Our conclusions are based on gene-level phenotypes: the reconstructed alleles were sufficient to confer growth after tryptophan depletion while still permitting growth in conditions that require the ancestral function. This establishes bifunctionality at the level of genes supporting 2 physiological requirements. While this does not quantify catalytic activities, prior literature provides strong biochemical precedent that both HisA and TrpA can acquire TrpF catalytic activity *in vitro* (Jürgens et al. 2000; Evran et al. 2012; Newton et al. 2017). See Supplementary Results and Discussion and Supplementary Fig. 10 for a speculative structure-based discussion for how the *hisA* and *trpA* mutations could encode bifunctional enzymes.

The severe tradeoffs observed in *hisA* paths compared to *trpA* paths highlight that the “generalist” route is not equally accessible to all enzymes. The decline in native *hisA* function likely reflects combined effects on catalytic properties and factors influencing total enzyme output *in vivo* (Newton et al. 2017). These differences provide an empirical basis for understanding how bifunctionality emerges during adaptation without gene duplication, and how evolution is shaped by the structure and frequency of environmental switching.

### Caveats and interpretation of “bifunctionality” at the gene level

A key point of framing is that our conclusions are based on gene-level phenotypes. The reconstructed *hisA* and *trpA* alleles were sufficient to confer growth after tryptophan depletion in Δ*trpF* strains, while still permitting growth under conditions requiring the ancestral functions. This establishes bifunctionality at the level of genes supporting 2 physiological requirements. It does not, by itself, quantify catalytic activities or identify the biochemical mechanism.

That said, several lines of evidence support the interpretation that the most parsimonious explanation is acquisition of TrpF-like catalytic activity by the mutant proteins while retaining native catalytic function, consistent with prior literature (see Fig. 1c for the reactions catalyzed by the 3 enzymes). First, HisA and TrpA variants that rescue Δ*trpF* in other studies have been shown to possess TrpF catalytic activity *in vitro* (Jürgens et al. 2000; Evran et al. 2012; Newton et al. 2017). Second, some HisA variants with TrpF catalytic activity retain measurable native activity *in vitro* (Jürgens et al. 2000; Newton et al. 2017). Third, in the present study, most *trpA* variants displayed wild-type growth when complemented with *trpF* (Fig. 4b), supporting retention of TrpA function *in vivo* for many alleles. Conversely, where complementation experiments indicated partial deficits, they are consistent with altered TrpA function rather than bypass of the pathway.

### Divergent mutational spectra between evolution experiments

It is worth noting that while the phenotypic rescue appeared more rapid or frequent in Experiment 2 than in experiment 1, this difference primarily reflects the total absence of the *trpA*(P62fs) duplication in experiment 1. The exact reason why this highly prevalent mutation never emerged in Experiment 1 remains unresolved, but it may stem from a downstream physiological effect of the different ancestral genotypes used. The ancestors in Experiment 1 carried *gal* mutations (Δ*P_gal_* Δ*galE*::*T_lux_*) to control phage sensitivity, whereas Experiment 2 utilized strains carrying the artificial gene transfer agent GTA22. It is possible that pleiotrophic effects in the *gal* background alter metabolic flux or increase the energetic and cellular cost of intermediate accumulation within the interrupted tryptophan pathway of a Δ*trpF* mutant. Such an effect could exert stronger early selection for inactivating mutations upstream in the pathway (mutations in *trpE* or *trpD* were very common; see Supplementary Results and Discussion). While disrupting the beginning of the pathway removes the cost of un-utilized flux, it effectively creates an evolutionary dead-end by preventing subsequent adaptive mutations from restoring full pathway function. Such a metabolic constraint may have biased the evolutionary trajectories of Experiment 1 away from *trpA*-based solutions, explaining the contrasting spectrum of rescue mechanisms observed between the 2 experiments.

### Conclusion

Our study provides empirical evidence that gene-level multifunctionality can repeatedly emerge during adaptation without the immediate necessity of gene duplication and divergence. By evolving Δ*trpF* populations under intermittent selection for restored prototrophy, we demonstrated that mutations in multiple genes (*hisA* and *trpA*) can restore growth in the absence of tryptophan while retaining ancestral functions. Crucially, these findings emphasize that the structure of selection—in this case, intermittent selection imposed by nutrient depletion—plays a defining role in shaping which evolutionary paths are accessible and successful within a native genomic and regulatory context. Ultimately, this work offers a broader framework for understanding how environmental switching promotes gene-level generalism, contrasting with models requiring gene duplication and divergence under continuous selection.

## Supplementary Material

jkag167_Supplementary_Data

## Data Availability

All genome re-sequencing data have been deposited in the Sequence Read Archive (SRA) with BioProject accession code PRJNA1092712. Supplemental material available at G3 online.

## Correspondence

Correspondence and requests for materials should be addressed to JN.
