# Supplementary material for: Experimental evolution reveals bifunctional genetic solutions to loss of trpF in Salmonella enterica
Source: G3 (Bethesda). 2026 Jul 6;16(10):jkag167. doi: 10.1093/g3journal/jkag167 (PMC13643763; doi:10.1093/g3journal/jkag167)
Supplement: Supplementary file 1 [file jkag167_supplementary_data.zip › Supplemental_Text_and_Figures_G3-2026-406697.pdf]

# Experimental evolution reveals bifunctional genetic solutions to loss of *trpF* in *Salmonella enterica*

Joakim Näsvall[1*] and Hind Abdalaal[1]

[1] Dept. of Medical Biochemistry and Microbiology, Uppsala University, Uppsala, Sweden
[*] Corresponding author. Email: joakim.nasvall@imbim.uu.se, jocke.nasvall@gmail.com

Supplementary Results and Discussion
Supplementary figures S1 – S10
Supplementary References

## Supplementary Results and Discussion

### Supplementing the growth medium with tryptophan causes growth defects that can be overridden by guanosine

The HisA variants with the most Δ*trpF* rescue activity displayed faster growth in the absence of tryptophan compared to its presence (Figures S3 and S4). This observation might be linked to the availability of phosphoribosylpyrophosphate (PRPP), a precursor molecule common to the *de novo* synthesis of histidine, tryptophan, purines, pyrimidines, and pyridine nucleotides.

In wild-type *Salmonella enterica*, histidine feedback-inhibits the first enzyme (HisG) of its biosynthetic pathway, making HisG the committing and rate-determining step (Winkler and Ramos-Montañez 2009). Conversely, a mutation in any other enzyme besides HisG is expected to decrease histidine synthesis, leading to increased flux into the pathway due to derepression of the operon and reduced HisG inhibition. Consequently, a HisA mutation could potentially increase PRPP consumption for histidine biosynthesis, limiting its availability for other pathways.

Tryptophan addition slightly reduces the PRPP pool in wild-type *S. enterica*, possibly through indirect effects on other pathways (Sadler and Switzer 1977). A similar phenomenon was observed in *S. enterica* mutants with a transposon insertion in *trpC*, exhibiting a filamentous growth phenotype. This phenotype was reversed by the addition of histidine, tryptophan, mutations in *hisF* or *hisH*, but not by nucleotides (Henry et al. 2005). This effect was attributed to excessive PRPP channelling into tryptophan synthesis, leaving insufficient PRPP for histidine biosynthesis.

We hypothesized that the observed growth inhibition of HisA mutants upon tryptophan addition might be related to its effect on the PRPP pool. Reduced PRPP synthesis due to tryptophan addition, coupled with increased PRPP channelling into the histidine pathway due to the HisA mutation, could potentially lead to insufficient nucleotide synthesis.

Given the critical role of purines (adenosine and guanosine) in energy homeostasis and as building blocks for RNA and DNA, compared to the less demanding pyrimidines (cytosine, uridine, and thymidine (Buckstein et al. 2008), we reasoned that if the growth reduction stemmed from limited PRPP availability, supplementing the medium with guanosine could rescue the growth defect. Notably, guanosine addition improved the growth of HisA mutants in tryptophan-containing medium but had no significant effect on wild-type HisA strains (including *trpA* mutants).

Regardless of the precise mechanism underlying the growth reduction in HisA mutants upon tryptophan addition, it appears unrelated to a direct limitation in histidine or tryptophan biosynthesis. Therefore, to isolate the specific effects on the native function of *hisA*, guanosine was added to the growth media when assaying the growth of reconstructed mutants in the presence of tryptophan (Figures 3, 4, S3 – S6, S8 – S9).

**Detailed analysis of unidentified growth-rescue mechanisms**
As noted in the main text, populations 1-7 and 2*mutS*22 achieved growth rescue through mechanisms that did not involve mutations in *hisA* or *trpA*. Identification of the specific mutations responsible for this phenotype proved challenging. While both populations shared mutation targets in *yafS*, *yeaG*, *trpD*, and large duplications between rRNA operons, these were present in other populations and deemed unlikely candidates for the growth-rescue phenotype.

The involvement of *trpD* was specifically excluded through genetic reconstruction:

- Reintroducing the wild-type allele of *trpD* into a clone from population 2*mutS*22 did not abolish the growth-rescue phenotype.
- The phenotype could not be transferred to the ancestral strain through generalized transduction, suggesting a polygenic basis where multiple subtle mutations contribute to the trait.

**Speculative structural interpretations for Δ*trpF* rescue**
This section provides structural considerations consistent with the observed ability of evolved alleles to support growth, though these are not intended as evidence for specific catalytic mechanisms.

The enzymes TrpF, HisA, and TrpA are structurally related $(\beta\alpha)_8$-barrel proteins that act on similar substrates. Both TrpF and HisA catalyze Amadori rearrangements on their amino aldose substrates to form amino ketoses (Figure S1). Their catalytic mechanisms involve protonation of the ring oxygen of the reacting ribose by a general acid (D176 in HisA, D126 in TrpF) and deprotonation of the ribose 2' carbon by a general base (D7 in HisA, C7 in TrpF; Figure S1d).

For the TrpA reaction, a base (D60) abstracts a proton from the indole ring nitrogen (N1), while another residue (E49) acts as both catalytic acid and base that protonates the C3 position of the indole ring and deprotonates the hydroxyl of the leaving group (Figure S1e). Both HisA and TrpA have flexible loops that close over the active site after substrate binding (Söderholm et al. 2015; Duran et al. 2024 Nov 2). In TrpA, loop dynamics are critical for allosteric regulation by TrpB (Duran et al. 2024 Nov 2). In HisA, loop closure coincides with

the rearrangement of the substrate into an extended product-like conformation (Söderholm et al. 2015).

Evran *et al.* (Evran et al. 2012) employed rational design to introduce putative catalytic residues from TrpF into TrpA, but only achieved TrpF activity after extensive mutagenesis and gene shuffling. Notably, their proposed essential mutations (F22C and L177D) were not sufficient for detectable TrpF activity. They identified mutations (e.g., **G61S, T24A**) that, according to their conclusions, allowed the engineered active site to catalyse the TrpF reaction. Additionally, they identified mutations (most prominently **G98C**) that they concluded were beneficial but not essential for TrpF activity. Unfortunately Evran et al. (Evran et al. 2012) did not reconstruct single mutations to test their individual effects, but instead relied on the presence or absence of mutations after gene shuffling for their conclusions.

In contrast, our evolutionary approach identified several single amino acid substitutions (**G98C**, A67T, **T24S**, D27Y, **G61S**, Y102H) that were sufficient for rescue of Δ*trpF*, without introducing residues previously proposed to be essential for TrpF catalysis by mutant TrpA. Our results suggest these mutations play a more significant role than previously thought, and that *trpA* mutations can support Δ*trpF* growth without mutations that exactly transplant the TrpF active site into TrpA.

Superposing the crystal structures of TrpA with its native substrate and TrpF with its product analog rCdRP places the carboxyl group of TrpA E49 and the sulfide group of TrpF C7 close to each other (Figure S10f). Interestingly, TrpA E49 is only 3Å from the C2' of rCdRP, placing it in a position that could, in principle, participate in chemistry analogous to that required for the TrpF reaction. The respective general acids are far from each other, and TrpA D60 does not appear to be appropriately positioned for acting as general acid on PRA. The absence of appropriately located potential general acids for the TrpF reaction in TrpA in the superposed structures may not necessarily be a relevant problem; in the structure of the bifunctional HisA ortholog PriA from *Mycobacterium tuberculosis* the product CdRP binds in a different orientation than in the TrpF structure, more resembling the binding of ProFAR in the active site of HisA (Figures S10d and S10e) (Due et al. 2011). This observation suggests the possibility that if PRA binds to the active site of TrpA, it does not necessarily bind in an orientation similar to its binding in TrpF (or PriA), but rather that it needs to bind in a conformation similar to the native TrpA substrate.

Several TrpA mutations that resulted in Δ*trpF* growth rescue map to positions close to the active site and could potentially affect the local structure or dynamics around the catalytic residues D60 or E49: G61S and P62S affect residues next to D60; Y102, close to D60, likely helps lock it in place for native catalysis, and the Y102H mutation may allow more flexibility or make the active site cavity larger; T24, D27, and A67 pack against each other at the interface between loop 1 and a helix at the end of loop 2. Mutations in these residues (T24S, D27Y, and A67T) may disrupt this interaction, leading to more flexibility of loop 2, perhaps allowing D60 to reach the ribose ring oxygen of PRA. G98 is close to E49, below the substrate binding pocket. Mutations at this position (G98C and G98S) add bulky side-chains that may restructure the active site to allow binding or catalysis of the larger TrpF substrate. One mutation (Q250R) maps to the outside surface far from the active site, making any

speculation about its effect difficult. This mutation, however did not result in a significant growth improvement and may simply have been fixed due to the high mutation rate in the mutator strain it appeared in.

In a previous evolution experiment testing the Innovation – Amplification – Divergence model for the evolution of new functions (Näsvall et al. 2012), we found two mutations in HisA that generated TrpF activity at the expense of all detectable HisA activity: "dup13-15" and L169R. The former results in a three-amino acid insertion in a flexible loop (loop 1) that closes the active site after substrate binding (Söderholm et al. 2015). The three extra amino acids make the loop more flexible, allowing the positively charged guanidino group of R18 to stabilize binding of the negatively charged TrpF substrate (PRA) but simultaneously displaces a tryptophan (W145) that is necessary for binding the native substrate (Newton et al. 2017). Similarly, the L169R mutation placed an arginyl residue appropriately to fulfil the analogous role from a position below the active site, abolishing the binding of the native substrate (Newton et al. 2017). In the current experiments, all evolutionary paths involving HisA mutations started with a single amino acid substitution, Q18R, placing an arginine in the same position as the dup13-15 mutation but without loop expansion. The Q18R mutation, partially characterized in previous experiments, displays less rescue of Δ*trpF* growth than dup13-15, although it retains near wild-type HisA function (Lundin et al. 2020). This suggests the guanidino group of the arginine at position 18 in Q18R can reach a similar position as in the dup13-15 mutation, but the absence of extra amino acids in the loop may prevent the arginine from reaching its optimal position. Interestingly, three additional HisA mutations that improved the Δ*trpF* growth rescue of the Q18R mutant were found in the vicinity of position 18: H17R, A22T, and R23L. Each of these mutations improved overall growth in the evolution experiments but negatively impacted HisA function (Figures 3, 4), suggesting the mutations enhanced the ability to substitute for TrpF *in vivo* at the expense of HisA function. If the normal-sized loop of the Q18R mutant is not flexible enough to allow the arginine to reach deeply into the active site for efficient PRA binding or catalysis, these other mutations may make the loop more flexible, allowing the arginine to reach its optimal position, potentially improving the ability to substitute for TrpF *in vivo*. Added flexibility due to the adjacent mutations could act analogously to the loop extension in the dup13-15 mutant, compromising the loop structure and negatively impacting native catalysis. Other mutations in HisA are more difficult to rationalize; V168 and F199 pack against each other in the hydrophobic core, and the mutations V168A and F199L likely destabilize the native structure, at least locally. Another mutation (A127G) is close to V168 and F199 but points into the barrel, forming part of the bottom of the active site cavity. These mutations may destabilize the structure, negatively impact enzyme activity or steady-state levels, but make the active site more flexible in accommodating the alternative substrate. Other mutations in HisA affect surface-exposed charged or polar residues (T52, K107, R131), changing them to other charged or polar amino acids (T52S, K107E, R131H). Two of these (T52S, R131H) had modest or insignificant impacts on HisA function (Figure 4) while improving growth after tryptophan depletion, suggesting a different mode of action than the other mutations.

The evolution of bifunctional HisA is an example of substrate ambiguity, where an enzyme evolves to accommodate two different substrates while continuing to catalyze its original reaction. The main challenge is enabling the binding of the new substrate without losing the ability to bind the original one. For TrpA to retain its native function while evolving to

substitute for TrpF, a case of catalytic promiscuity, seems less likely at first glance. Not only must it bind a new substrate and catalyze an entirely different reaction, but it must also do so without losing its original function, which appears to be a very difficult problem.

However, arguments can be made that the mutant TrpA variants characterized by Evran *et al*. (Evran et al. 2012) were already bifunctional. *In vivo*, TrpA depends on TrpB for activity (Pittard and Yang 2008), and as observed by us, this also applies to the detectable rescue of a *trpF* mutant. In their selections and growth rate measurements Evran *et al*. expressed the mutant *trpA* alleles from multicopy plasmids, leading to competition for TrpB by the two TrpA variants. If the expression of plasmid-borne TrpA was sufficiently high, the mutant TrpA would outcompete the wild-type TrpA. If the mutant TrpA variants lacked TrpA activity, rescuing a *trpF* mutant would not be possible. Alternatively, though perhaps less likely, to gain TrpF activity in their selection, TrpA may have first acquired mutations that prevented association with TrpB and allowed it to function efficiently without its partner.

**Additional mutations potentially affecting fitness in evolution experiments**
Beyond primary causative mutations, many lineages harboured adaptations to the laboratory environment:
**General fitness:** Mutations in *pykF*, *rpoS*, and *spoT* are known to provide selective advantages in minimal glucose media (King et al. 2004; Phillips et al. 2016; Knöppel et al. 2018).

**Metabolic and regulatory adjustments:** Mutations in *hisG* co-occurred with *trpA* mutations in several populations, potentially reducing wasteful consumption of PRPP (See "Supplementing the growth medium with tryptophan causes growth defects that can be overridden by guanosine" above).

**Operon regulation:** Mutations reducing the levels or function of $tRNA^{His}$ mimic histidine starvation and cause de-repression of *his* operon transcription (Winkler and Ramos-Montañez 2009). Several populations (1-1, 1-3, 2*mutS*3) had mutations in the gene (*hisR*) encoding $tRNA^{His}$ or its promoter (Tables S3, S6). These likely cause increased expression of the *his* operon, thereby increasing the steady-state levels of the evolving HisA protein.

**Evolutionary dead-ends:** A 98-bp deletion in *trpE* found in a non-growing population likely represents a mutation that provides a local fitness benefit (by stopping flux into the broken trp pathway) but prevents further adaptation toward tryptophan prototrophy.

## Supplementary Figures

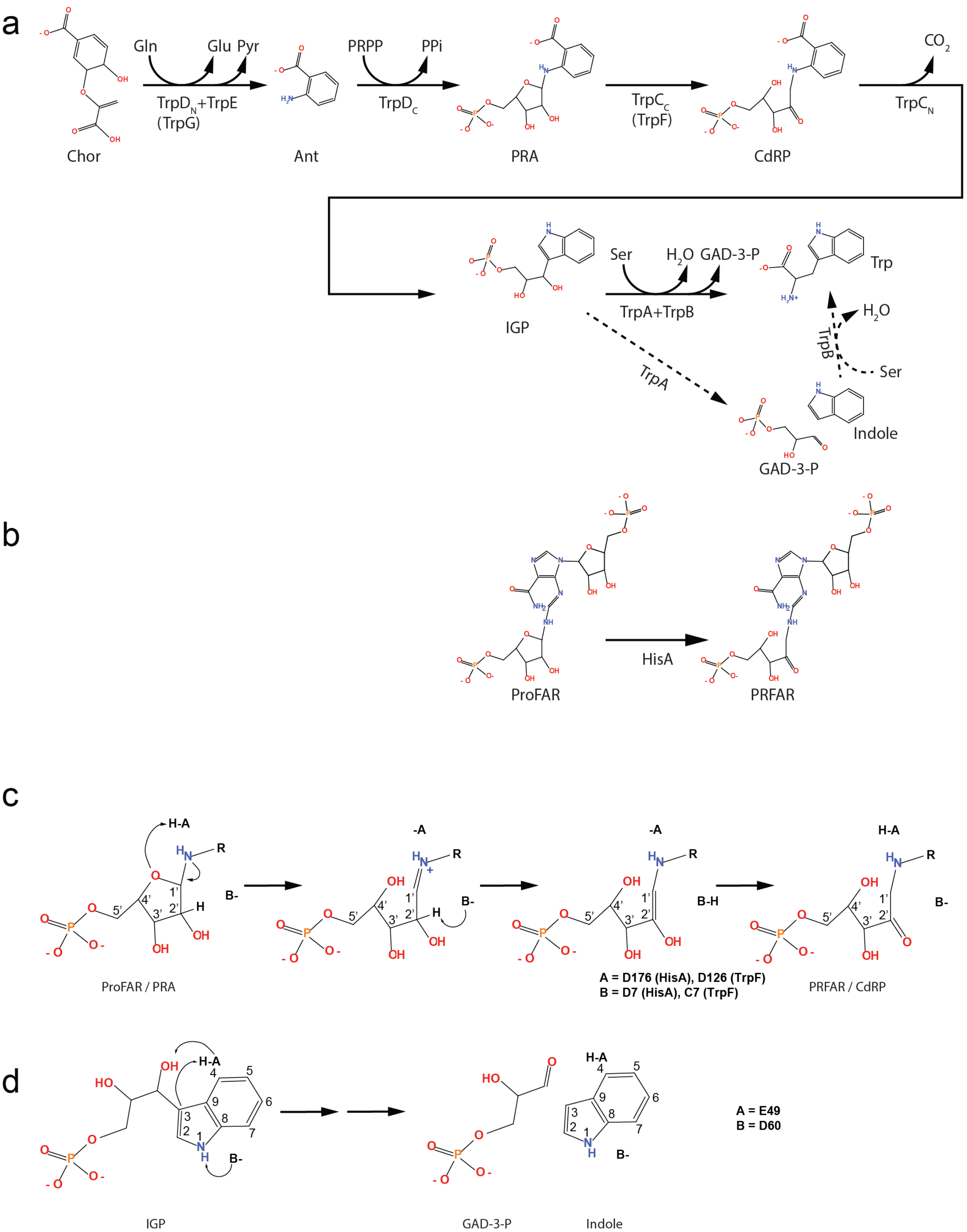


**Figure S1. (a)** Biosynthesis of tryptophan is catalyzed by the enzymes indicated below the reaction arrows. Subscript letters (N or C) refer to which domain of a fused protein (in *Salmonella* and other members of *Enterobacteriaceae*) contain the indicated activity, and protein names in parenthesis are the names of the corresponding proteins in organisms where the enzymes are not fused. For simplicity, we refer to the enzyme PRA isomerase contained within the C-terminal domain of TrpC as TrpF, despite it not being a separate protein in *Salmonella*. Gln, L-glutamine; Glu, L-glutamate; PRPP, phosphoribosyl-pyrophosphate; PPi, inorganic phosphate; $CO_2$, carbon dioxide; Ser, L-serine; GAD-3-P, glyceraldehyde-3-phosphate; Chor, chorismate; Ant,

anthranilate; PRA, phosphoribosyl anthranilate; CdRP, 1-(2-carboxyphenylamino)-1'-deoxyribulose-5'-phosphate; IGP, indole-3-glycerol phosphate; Trp, L-tryptophan. Dashed reaction arrows: while the physiological activity of tryptophan synthase (TrpA+TrpB) is the direct conversion of IGP to Trp (without the release of Indole as an intermediate), *in vitro* the reaction can be broken up into two separate reactions. **(b)** HisA (ProFAR isomerase) catalyzes the isomerization of N'-[(5'-phosphoribosyl)-formimino]-5-aminoimidazole-4-carboxamide ribonucleotide (ProFAR) to N'-[(5'-phosphoribulosyl) formimino]-5-aminoimidazole-4-carboxamide-ribonucleotide (PRFAR), the fourth step in histidine biosynthesis. **(c – d)** Part of the catalytic mechanisms for HisA, TrpF and TrpA. **(c)** The reactions catalyzed by HisA and TrpF. The general acid (D176 in HisA, D126 in TrpF) protonates the ribose ring oxygen, and the general base (D7 in HisA, C7 in TrpF) abstracts a proton from the C2' of the ribose. **(d)** The reaction catalyzed by TrpA. The general acid (D60) protonates the C3 carbon, and the general base (E49) abstracts a proton from the N1 nitrogen of the indole.

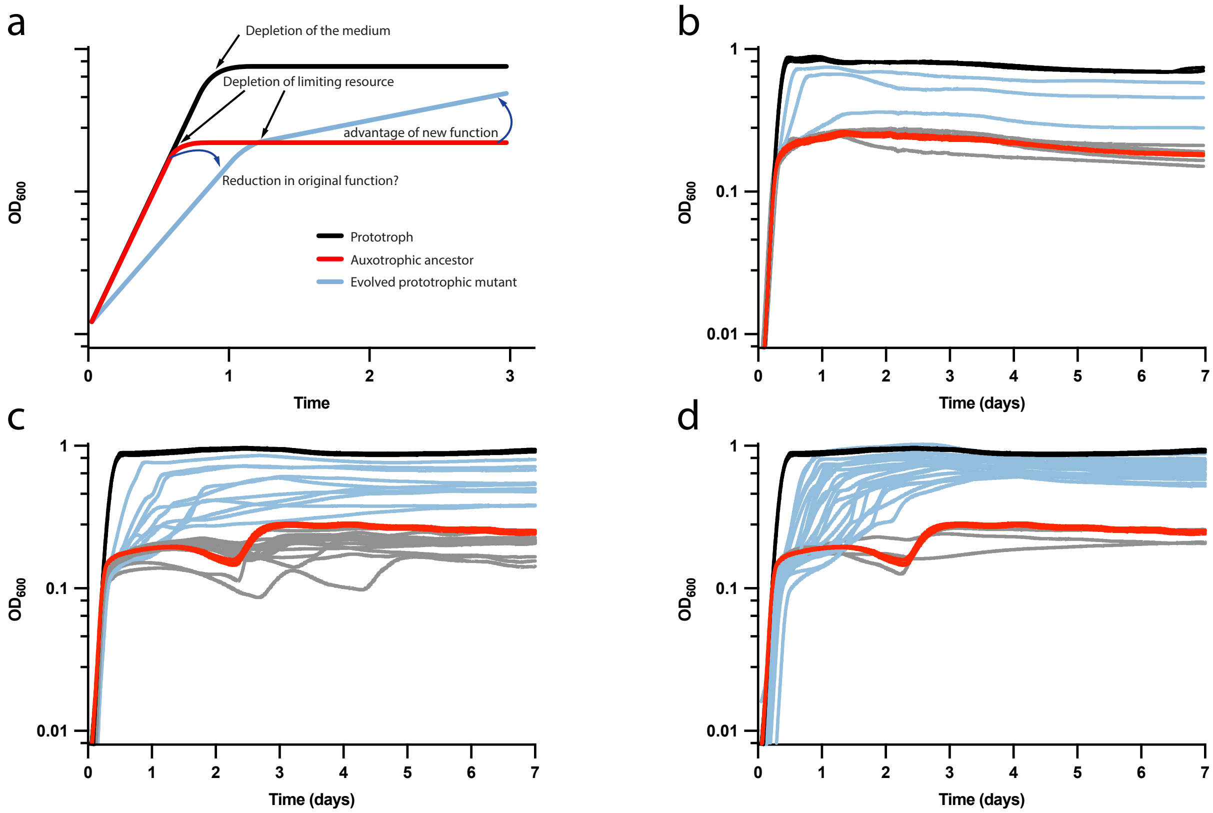


**Figure S2.** Growth Dynamics of Prototrophic and Auxotrophic Strains under Limiting Resources. **(a)** Hypothetical growth curves: the expected growth behaviour of a prototrophic strain (black), an auxotrophic strain (red), and an evolving prototroph (blue) under limiting resource conditions. The auxotroph (red) is expected to grow until the limiting resource is depleted and enter stationary phase at low population density. The prototroph (black) can continue growing until depletion of other essential nutrients or changes in medium composition (e.g., pH or metabolite accumulation) halt growth. The evolved prototroph (blue) exhibits growth after the limiting resource depletion, with a rate dependent on the rate of its newly acquired activity. If the original enzyme function is crucial for growth, reduced activity due to a mutation might lead to a slower growth rate before the limiting resource is depleted. **(b – d)** Batch growth curves of a tryptophan prototroph (*trpF*+, black), the ancestral tryptophan auxotroph (Δ*trpF*, red), the evolving populations from the final time-point that gained tryptophan independent growth (blue), and those that did not (grey). **(b)** The eight lineages from the first experiment. **(c)** The 24 non-mutator lineages from the second experiment. **(d)** The 24 mutator lineages from the second experiment (compared to the ancestral non-mutator strain).

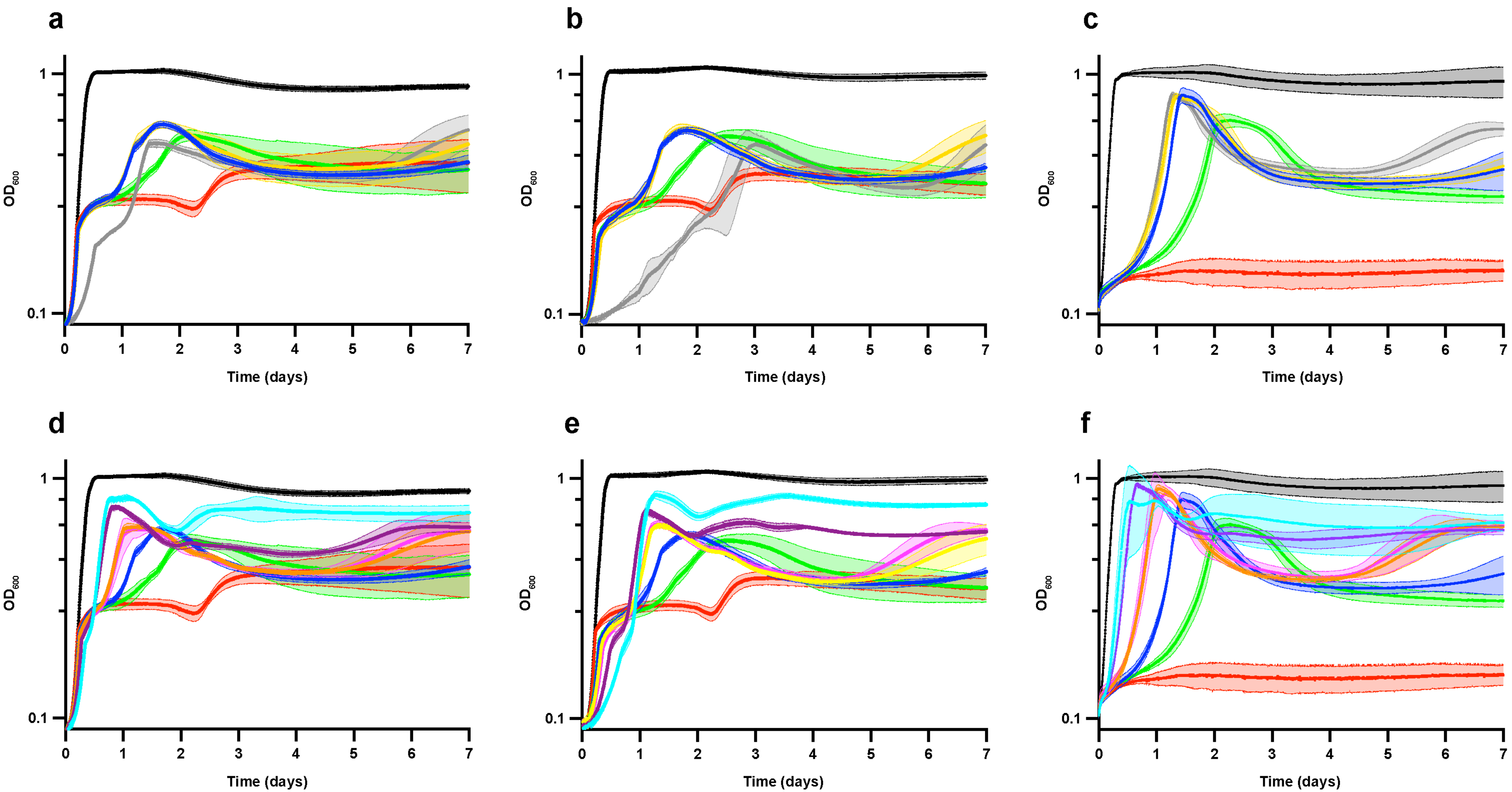


**Figure S3.** Growth of re-constructed *hisA* mutants. In all graphs the ancestral (*ΔtrpF*; red) and a wild-type (*trpF+*; black) strain is included for comparison. The curves show the average $OD_{600}$ with standard deviation of at least four biological replicates. **(a, d)** Cultures grown after 1000x dilution in 2x M9 + 0.4 % glucose supplemented with 5 µM tryptophan + 3 mM guanosine. **(b, e)** Cultures grown after 1000x dilution in 2x M9 + 0.4 % glucose supplemented with 5 µM tryptophan (no guanosine). **(c, f)** Cultures grown after 100x dilution in 2x M9 + 0.4 % glucose without supplementation. **(a - c)** *hisA* mutations found at 120 and 170 generations in population 1-1. Green; *hisA*(Q18R), grey; *hisA*(H17R Q18R), yellow; *hisA*(Q18R A22T), blue; *hisA*(Q18R K107E). **(d - f)** *hisA* mutations found at 320 and 420 generations in population 1-1. Green; *hisA*(Q18R; included for comparison), blue; *hisA*(Q18R K107E), pink; *hisA*(Q18R I62M K107E); orange; *hisA*(Q18R K107E V168A), purple; *hisA*(Q18R R23L K107E V168A), cyan; *hisA*(Q18R A22T R23L K107E V168A).

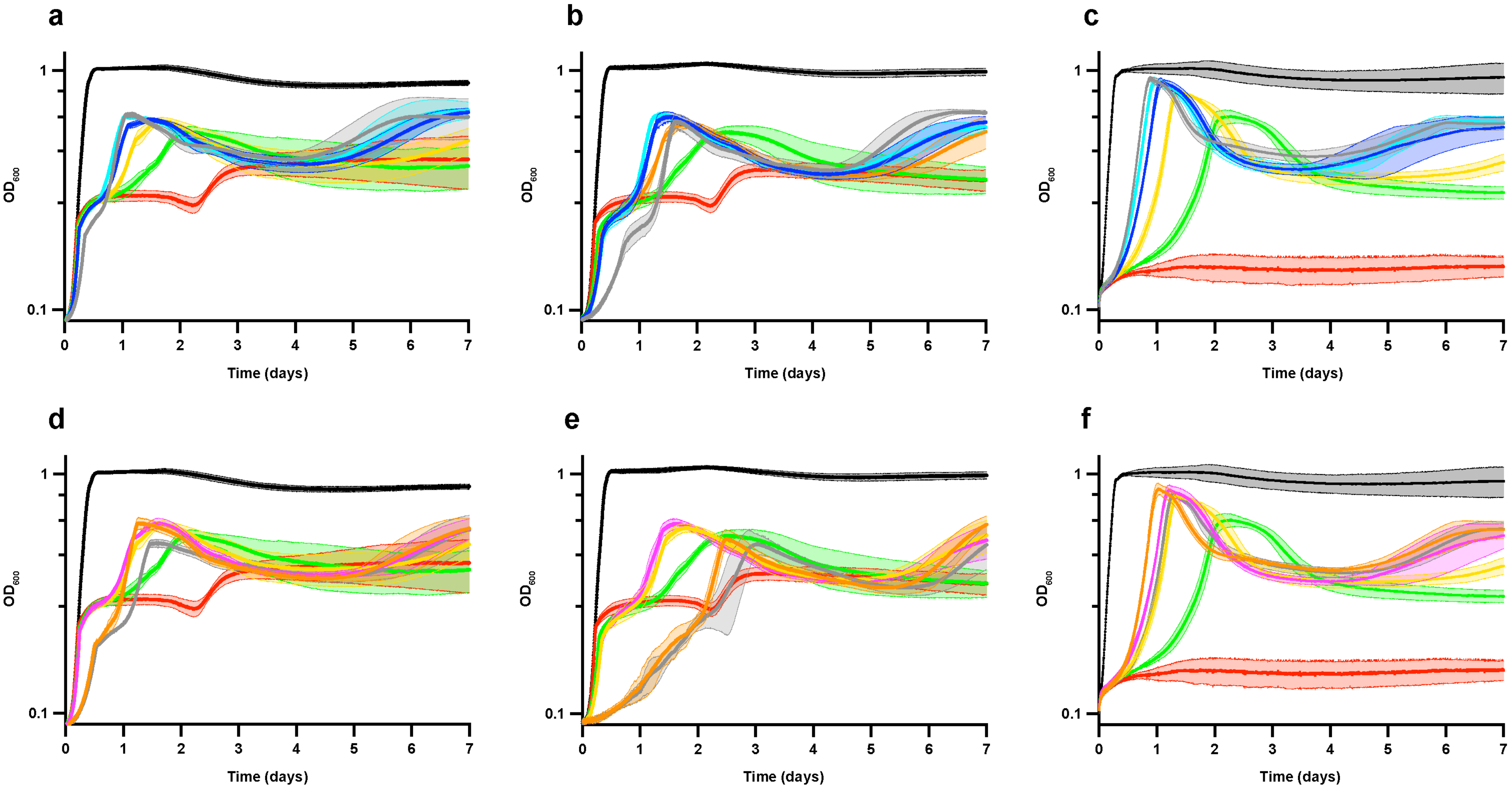


**Figure S4.** Growth of re-constructed *hisA* mutants, continued. In all graphs the ancestral (*ΔtrpF*; red) and a wild-type (*trpF*+; black) strain is included for comparison. The curves show the average $OD_{600}$ with standard deviation of at least four biological replicates **(a, d)** Cultures grown after 1000x dilution in 2x M9 + 0.4 % glucose supplemented with 5 µM tryptophan + 3 mM guanosine. **(b, e)** Cultures grown after 1000x dilution in 2x M9 + 0.4 % glucose supplemented with 5 µM tryptophan (no guanosine). **(c, f)** Cultures grown after 1000x dilution in 2x M9 + 0.4 % glucose without supplementation. **(a - c)** *hisA* mutations found in population 1-3. Green; *hisA*(Q18R), yellow; *hisA*(Q18R A22T), cyan; *hisA*(Q18R A22T V168A), grey; *hisA*(Q18R A22T I62M R131H), blue; *hisA*(Q18R A22T V168A F199L). **(d - f)** *hisA* mutations found in the *mutS* populations. Green; *hisA*(Q18R), yellow; *hisA*(Q18R A22T), orange; *hisA*(Q18R A127G), grey; *hisA*(H17R Q18R), pink; *hisA*(Q18R T52S).

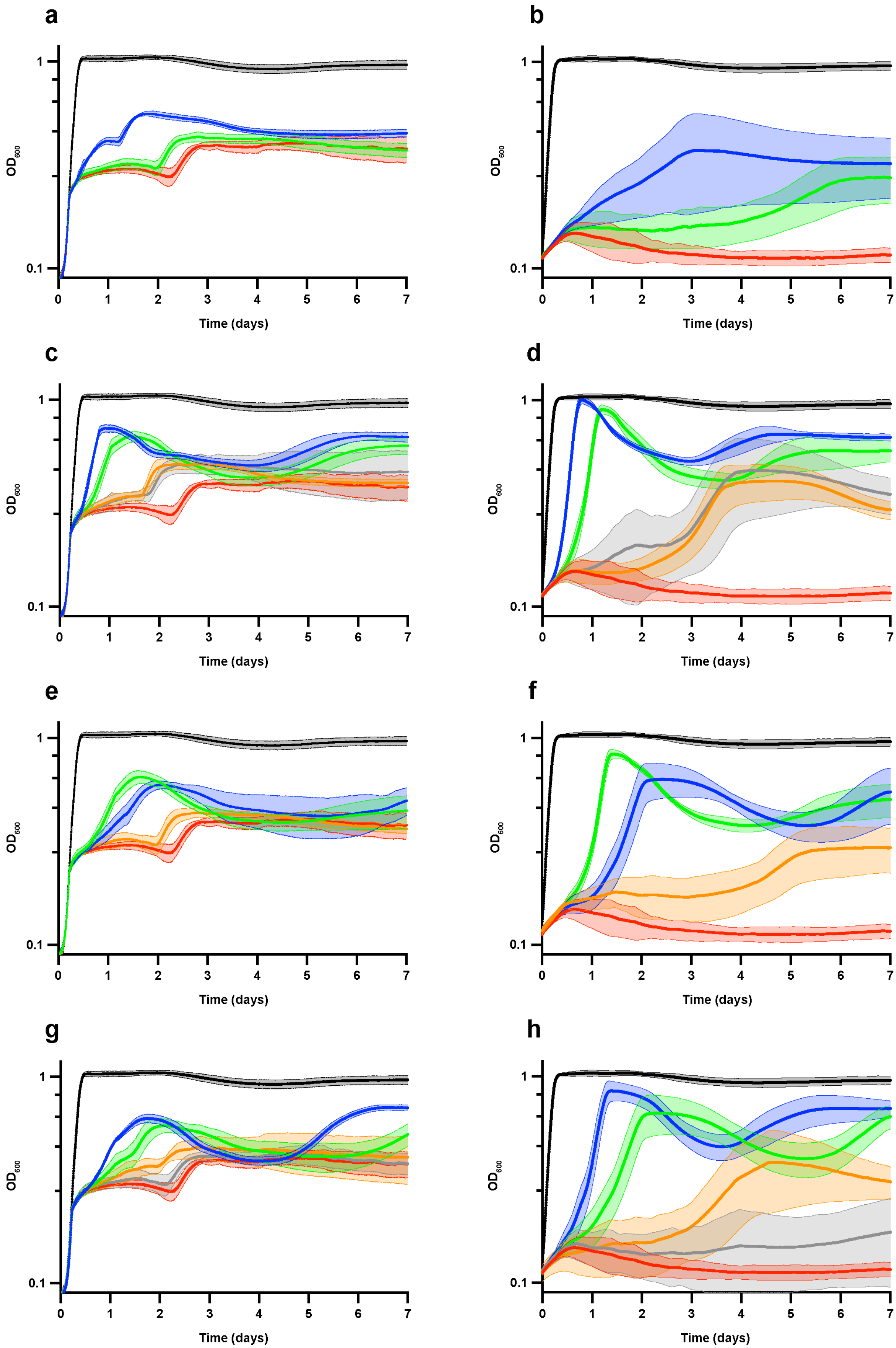

a
b
c
d
e
f
g
h
$OD_{600}$
1
0.1
0
1
2
3
4
5
6
7
Time (days)

**Figure S5.** Growth of re-constructed *trpA* mutants. In all graphs the ancestral (*ΔtrpF*; red) and a wild-type (*trpF*+; black) strain is included for comparison. The curves show the average $OD_{600}$ with standard deviation of five biological replicates. **(a, c, e, g)** Cultures grown after a 1000x dilution in 2x M9 + 0.4% glucose supplemented with 5 µM tryptophan + 3 mM guanosine. **(b, d, f, h)** Cultures grown after a 100x dilution in 2x M9 + 0.4% glucose without supplementation. **(a, b)** *trpA* mutations found in the non-mutator populations. Green; *trpA*(A67T), blue; *trpA*(P62fs). **(c, d)** *trpA* mutations found in mutator populations 2*mutS*4, 2*mutS*11, and 2*mutS*23. Grey; *trpA*(D27Y), orange; *trpA*(G98C), green; *trpA*(D27Y G98S), blue; *trpA*(D27Y G98C). (e, f) *trpA* mutations found in mutator population 2*mutS*14. Orange; *trpA*(Y102H), blue; *trpA*(G98S Y102H), green; *trpA*(G98S Y102H Q250R). (g, h) *trpA* mutations found in mutator population 2*mutS*16. Grey; *trpA*(T24S), orange; *trpA*(G61S), green; *trpA*(T24S G61S), blue; *trpA*(T24S G61S P62S).

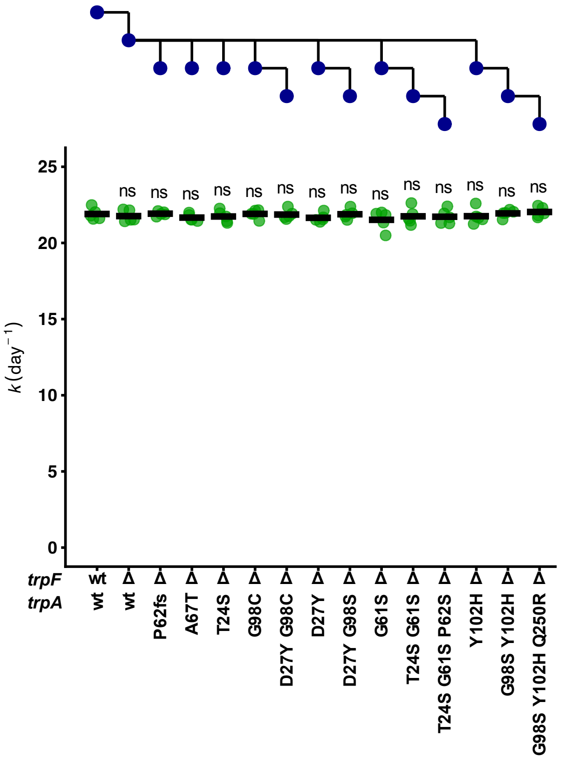


**Figure S6.** Growth rates of *trpA* mutants prior to tryptophan depletion. Exponential growth rates were determined in the early rapid growth phase ($0.095 > OD_{600} < 0.184$) in M9 glucose medium supplemented with tryptophan (5 µM) and guanosine (3 mM), and set relative to a *trpF*(wt) *trpA*(wt) strain grown in the same experiment. Black bars represent averages. The lineage map above the plot indicates the order of appearance of each mutation as deduced from sequencing of isolated clones from various time-points in the evolving populations. ns, non-significant ($p > 0.05$) based on a Welch's t-test, corrected for multiple testing using the Benjamini-Hochberg method. Each mutant was compared to its immediate ancestor, as indicated in the lineage maps. All strains were grown in at least four independent biological replicates. All strains were grown as five biological replicates.

a

*trpA*(wt)

```
            60 61 62
UCCGAUCCGCUGGCCGAUGGCCCUACCAUCCAGAAUGCGAACUUACGCGCCUUCGCCGCUGGCGUCACGCCGGCUCAGUGUUUUGAAAUG
 S  D  P  L  A  D  G  P  T  I  Q  N  A  N  L  R  A  F  A  A  G  V  T  P  A  Q  C  F  E  M
```

b

*trpA*(P62fs)

```
            60 61       62
UCCGAUCCGCUGGCCGAUGGCCGAUGGCCCUACCAUCCAGAAUGCGAACUUACGCGCCUUCGCCGCUGGCGUCACGCCGGCUCAGUGUUUUGAAAUG
 S  D  P  L  A  D  G  R  W  P  Y  H  P  E  C  E  L  T  R  L  R  R  W  R  H  A  G  S  V  F  *
  P  I  R  W  P  M  A  D  G  P  T  I  Q  N  A  N  L  R  A  F  A  A  G  V  T  P  A  Q  C  F  E  M
```

c

*trpA*(61-62insRW)

```
            60 61      62
UCCGAUCCGCUGGCCGAUGGCCGAUGGCCUACCAUCCAGAAUGCGAACUUACGCGCCUUCGCCGCUGGCGUCACGCCGGCUCAGUGUUUUGAAAUG
 S  D  P  L  A  D  G  R  W  P  T  I  Q  N  A  N  L  R  A  F  A  A  G  V  T  P  A  Q  C  F  E  M
```

**Figure S7.** A frameshift mutation in *trpA* generates a bifunctional gene. **(a)** Codons 55 – 84 in wild-type *trpA*. Top, mRNA sequence; bottom, amino acid sequence. Bold text: five-nucleotide repeats (UGGCC) involved in generation of the P62fs mutant. Blue box: The 7 bp sequence (UGGCCGA) that is duplicated in the P62fs mutant. **(b)** The corresponding sequence in the *trpA*(P62fs) mutant. The underlined quadruplet (CCCU) is a potential "slippery" site, where a ribosome with peptidyl-tRNA$^{Pro}$ in its P-site could "slip" one base forward and continue translating in the +1 reading frame, resulting in the insertion of two extra amino acids (arg-trp) compared to the wild-type sequence. **(c)** The corresponding sequence in the 61-62insRW mutant generated to test the hypothetical frameshift suppression mechanism. Black text in amino acid sequence: wild-type TrpA sequence. Red text in amino acid sequence: mis-incorporated amino acids if translation continues in the original (zero) reading frame. Blue text in amino acid sequence: mis-incorporated amino acids if translation shifts into the +1 frame. Red text in mRNA: A UGA stop codon encountered in the zero frame in the Pro62fs mutant. The amino acid at position 60 (D60) is one of the catalytic residues for the native reaction, probably limiting the window of functional suppression to somewhere after D60 and before the zero frame stop codon.

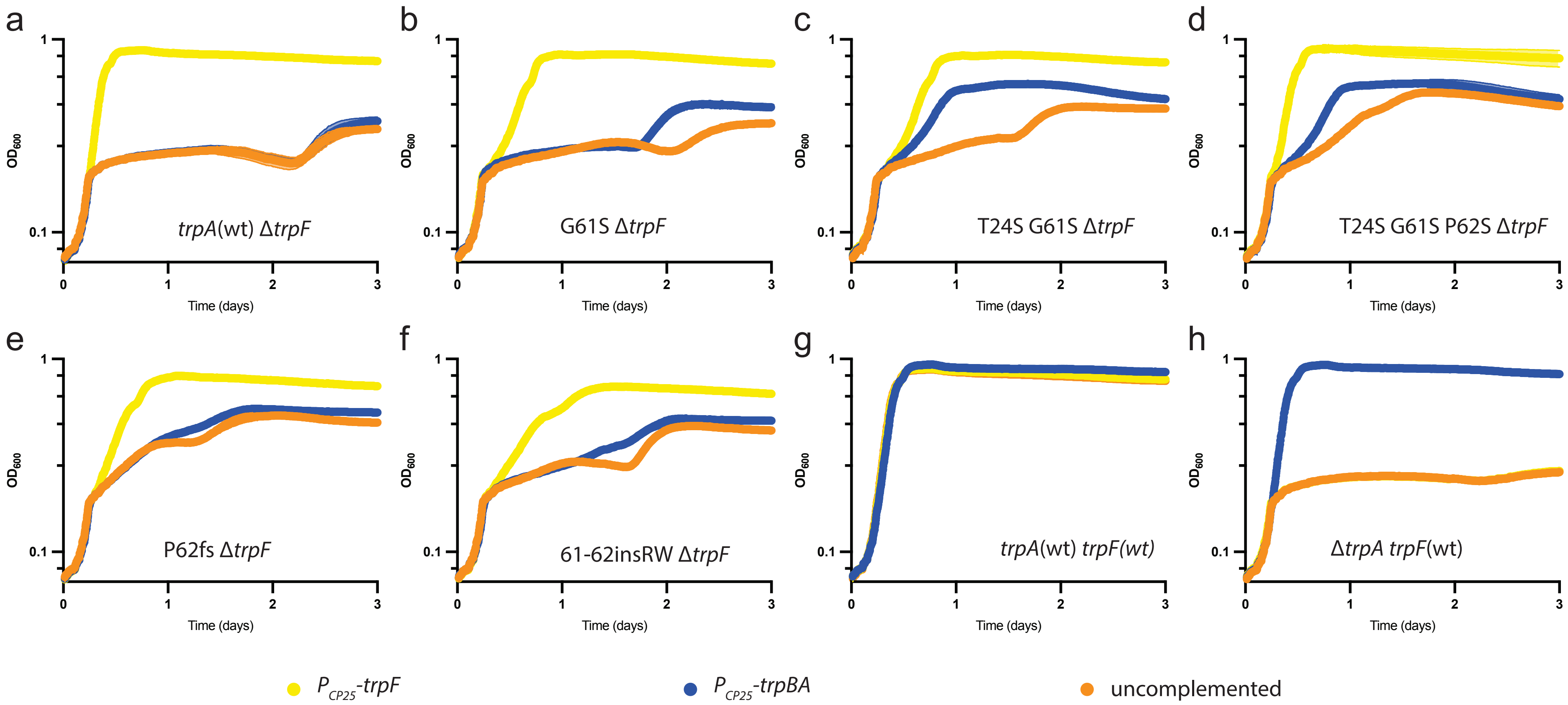


**Figure S8.** Growth of complemented *trpA* mutants. The curves show the average $OD_{600}$ with standard deviation of five biological replicates (most of the error ranges are too small to be seen). To reduce noise from a faulty Bioscreen machine, the data was smoothed by taking the median in an eleven data point sliding window. All cultures were grown after a 1000x dilution in 2x M9 + 0.4% glucose supplemented with 5 µM tryptophan. **(a)** *trpA*+ Δ*trpF* (ancestor for the evolution experiments) **(b)** *trpA*[G61S] Δ*trpF* **(c)** *trpA*[T24S G61S] Δ*trpF* **(d)** *trpA*[T24S G61S P62S] Δ*trpF*. **(e)** *trpA*[P62fs] Δ*trpF* **(f)** *trpA*(61-62insRW), a mutant constructed to test a hypothetical frameshift suppression mechanism **(g)** wild-type (*trpA*+ *trpF*+) **(h)** Δ*trpA trpF*+. Orange; uncomplemented strains, blue; strains complemented with *trpBA*; yellow; strains complemented with *trpF*.

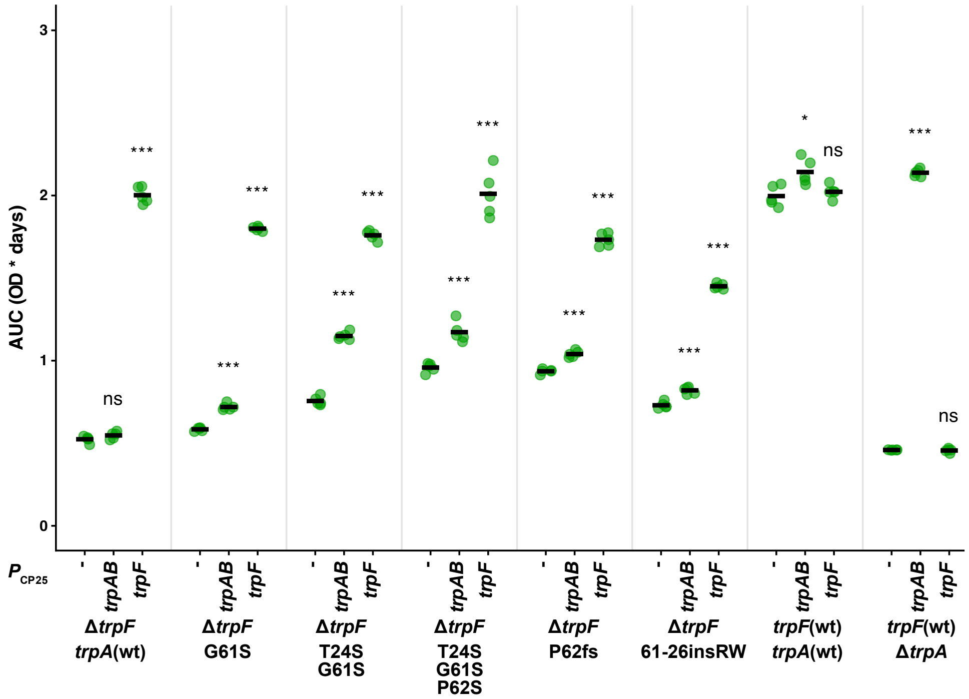


**Figure S9.** Growth of complemented *trpA* mutants. Growth is reported as the area under the curve (AUC) for the first 3 days. Black bars represent averages. ns, non-significant ($p > 0.05$); *, $p < 0.05$; **, $p < 0.01$; ***, $p < 0.001$, based on a Welch's t-test, corrected for multiple testing using the Benjamini-Hochberg method. Each complemented ($P_{CP25}$-*trpF* or $P_{CP25}$-*trpAB*) strain was compared to the isogenic, non-complemented (-) strain. All strains were grown in five independent biological replicates.

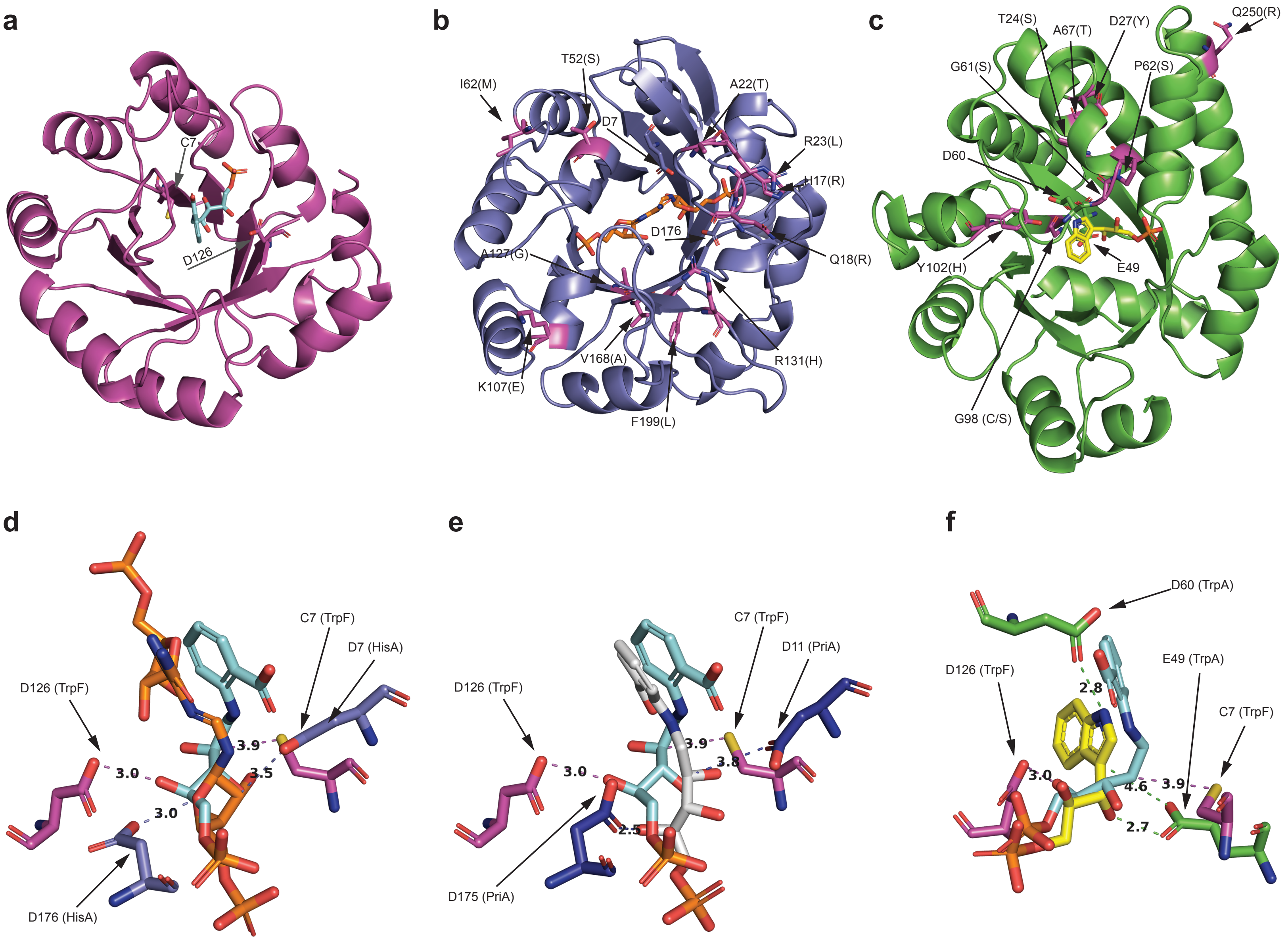

a
C7
D126
b
I62(M)
T52(S)
D7
A22(T)
R23(L)
H17(R)
D176
Q18(R)
A127(G)
V168(A)
R131(H)
K107(E)
F199(L)
c
T24(S)
A67(T)
D27(Y)
Q250(R)
G61(S)
P62(S)
D60
Y102(H)
E49
G98 (C/S)
d
C7 (TrpF)
D7 (HisA)
D126 (TrpF)
3.9
3.0
3.5
3.0
D176 (HisA)
e
C7 (TrpF)
D11 (PriA)
D126 (TrpF)
3.9
3.0
3.8
2.5
D175 (PriA)
f
D60 (TrpA)
D126 (TrpF)
E49 (TrpA)
2.8
C7 (TrpF)
3.0
4.6
3.9
2.7

**Figure S10.** Crystal structures of **(a)** *Thermotoga maritima* TrpF (pdb code: 1lbm) with bound product analogue rCdRP (cyan C-atoms), **(b)** *S. enterica* HisA (D7N D176A; pdb code: 5a5w) with bound substrate ProFAR (orange C-atoms), *in silico*-reverted to wild-type sequence to show the likely positions of the general acid and base, and **(c)** *S. enterica* TrpA (D60N; pdb code: 1a5b) with bound substrate IGP (yellow C-atoms), *in silico*-reverted to wild-type sequence. **(d)** Superposed HisA and TrpF, showing only the locations of the ligands and the general acids and bases, and the distances between the catalytic residues and reacting atoms. **(e)** Same as in (d) but TrpF and *Mycobacterium tuberculosis* PriA (pdb code: 2y85) with bound substrate analogue rCdRP (residues from PriA with dark blue C-atoms, and rCdRP with light grey C-atoms). **(f)** Same as (d) but TrpA and TrpF. The locations of mutations in HisA (b) and TrpA (c) are shown with magenta C-atoms and magenta cartoon. Arrows point at catalytic residues and mutant positions, with wild-type residues before parentheses and mutant residues within parentheses. The images and *in silico* mutagenesis were made with PyMOL (Schrödinger, LLC 2021). PriA and HisA were superposed in PyMOL using 'align' (RMSD = 1.335 over 1003 atoms), TrpA and TrpF were superposed on HisA using 'cealign' (RMSD = 4.888 and 4.593 over 176 residues for TrpF:HisA and TrpA:HisA, respectively).

## Supplementary References


Buckstein MH, He J, Rubin H. 2008. Characterization of Nucleotide Pools as a Function of Physiological State in Escherichia coli. J Bacteriol. 190(2):718–726. https://doi.org/10.1128/jb.01020-07

Due AV et al. 2011. Bisubstrate specificity in histidine/tryptophan biosynthesis isomerase from Mycobacterium tuberculosis by active site metamorphosis. Proc Natl Acad Sci U S A. 108(9):3554–3559. https://doi.org/10.1073/pnas.1015996108

Duran C et al. 2024. Altering Active-Site Loop Dynamics Enhances Standalone Activity of the Tryptophan Synthase Alpha Subunit. ACS Catal. 16986–16995. https://doi.org/10.1021/acscatal.4c04587

Evran S, Telefoncu A, Sterner R. 2012. Directed evolution of (βα)8-barrel enzymes: establishing phosphoribosylanthranilate isomerisation activity on the scaffold of the tryptophan synthase α-subunit. Protein Eng Des Sel. 25(6):285–293. https://doi.org/10.1093/protein/gzs015

Henry T, García-del Portillo F, Gorvel JP. 2005. Identification of Salmonella functions critical for bacterial cell division within eukaryotic cells. Mol Microbiol. 56(1):252–267. https://doi.org/10.1111/j.1365-2958.2005.04540.x

King T, Ishihama A, Kori A, Ferenci T. 2004. A regulatory trade-off as a source of strain variation in the species Escherichia coli. J Bacteriol. 186(17):5614–5620. https://doi.org/10.1128/JB.186.17.5614-5620.2004

Knöppel A et al. 2018. Genetic Adaptation to Growth Under Laboratory Conditions in Escherichia coli and Salmonella enterica. Front Microbiol. 9:756. https://doi.org/10.3389/fmicb.2018.00756

Lundin E, Näsvall J, Andersson DI. 2020. Mutational Pathways and Trade-Offs Between HisA and TrpF Functions: Implications for Evolution via Gene Duplication and Divergence. Front Microbiol. 11:588235. https://doi.org/10.3389/fmicb.2020.588235

Näsvall J, Sun L, Roth JR, Andersson DI. 2012. Real-time evolution of new genes by innovation, amplification, and divergence. Science. 338(6105):384–387. https://doi.org/10.1126/science.1226521

Newton MS et al. 2017. Structural and functional innovations in the real-time evolution of new (βα)8 barrel enzymes. Proc Natl Acad Sci U S A. 114(18):4727–4732. https://doi.org/10.1073/pnas.1618552114

Phillips KN, Castillo G, Wünsche A, Cooper TF. 2016. Adaptation of Escherichia coli to glucose promotes evolvability in lactose. Evolution. 70(2):465–470. https://doi.org/10.1111/evo.12849

Pittard J, Yang J. 2008. Biosynthesis of the Aromatic Amino Acids. EcoSal Plus. 3(1):10.1128/ecosalplus.3.6.1.8. https://doi.org/10.1128/ecosalplus.3.6.1.8

Sadler WC, Switzer RL. 1977. Regulation of Salmonella phosphoribosylpyrophosphate synthetase activity in vivo. Deductions from pool measurements. J Biol Chem. 252(23):8504–8511. https://doi.org/10.1016/S0021-9258(19)75248-X

Schrödinger, LLC. 2021. The PyMOL Molecular Graphics System, Version 2.5.0.

Söderholm A et al. 2015. Two-step Ligand Binding in a (βα)8 Barrel Enzyme: SUBSTRATE-BOUND STRUCTURES SHED NEW LIGHT ON THE CATALYTIC CYCLE OF HisA. J Biol Chem. 290(41):24657–24668. https://doi.org/10.1074/jbc.M115.678086

Winkler ME, Ramos-Montañez S. 2009. Biosynthesis of Histidine. EcoSal Plus. 3(2):10.1128/ecosalplus.3.6.1.9. https://doi.org/10.1128/ecosalplus.3.6.1.9
